# Supplementary material for: Constitutive activation of the PI3K-Akt-mTORC1 pathway sustains the m.3243 A > G mtDNA mutation
Source: Nat Commun. 2021 Nov 4;12:6409. doi: 10.1038/s41467-021-26746-2 (PMC8568893; doi:10.1038/s41467-021-26746-2)
Supplement: Supplementary file 1 — Supplementary Information [file 41467_2021_26746_MOESM1_ESM.pdf]

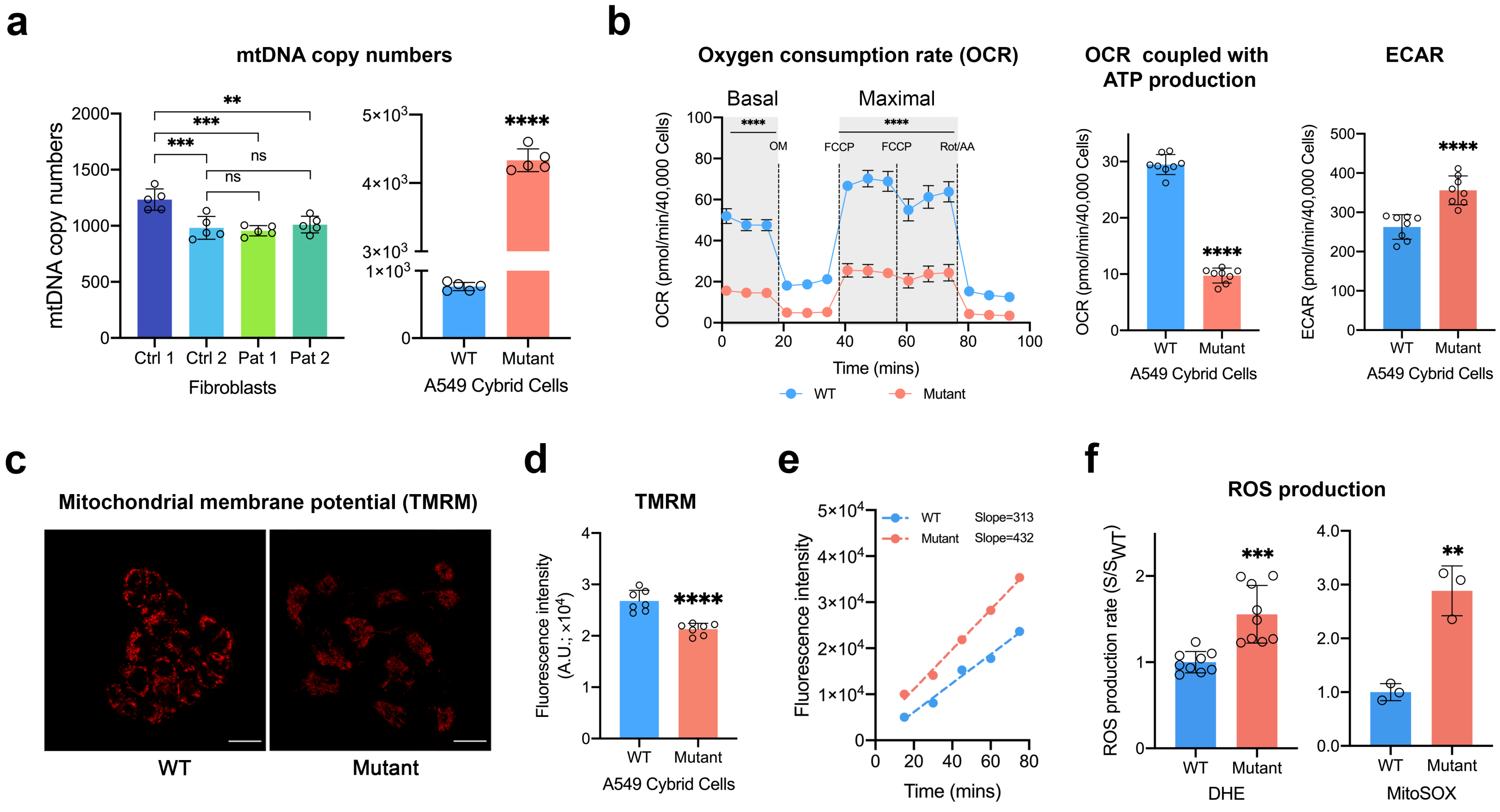
a
mtDNA copy numbers
mtDNA copy numbers
2000
1500
1000
500
0
**
***
***
ns
ns
Ctrl 1
Ctrl 2
Pat 1
Pat 2
Fibroblasts
5×10^3
4×10^3
3×10^3
1×10^3
0
****
WT
Mutant
A549 Cybrid Cells
b
Oxygen consumption rate (OCR)
Basal
Maximal
OCR (pmol/min/40,000 Cells)
****
OM
FCCP
FCCP
Rot/AA
Time (mins)
WT
Mutant
OCR coupled with ATP production
OCR (pmol/min/40,000 Cells)
****
WT
Mutant
A549 Cybrid Cells
ECAR
ECAR (pmol/min/40,000 Cells)
****
WT
Mutant
A549 Cybrid Cells
c
Mitochondrial membrane potential (TMRM)
WT
Mutant
d
TMRM
Fluorescence intensity (A.U.; ×10^4)
****
WT
Mutant
A549 Cybrid Cells
e
Fluorescence intensity
WT Slope=313
Mutant Slope=432
Time (mins)
f
ROS production
ROS production rate (S/S_WT)
***
WT
Mutant
DHE
**
WT
Mutant
MitoSOX

Supplementary Fig. 1. Mitochondrial dysfunction and increased ROS generation are also found in the A549 cybrid cells.

a Mitochondrial DNA copy number of fibroblasts (p = 0.0002) and A549 cybrid cells (p < 0.0001; n = 5 independent biological samples for all cell lines).

b Cell respiratory capacity was also measured using the Seahorse XFe96 extracellular flux analyser in A549 cybrid cells (p < 0.0001; n = 8 culture wells, further normalised to mtDNA copy number) showing a major decrease in oxygen consumption under all conditions. Oxygen consumption dependent on ATP production and ECAR are plotted.

c-d The mitochondrial membrane potential of A549 cybrid cells measured using TMRM with confocal imaging (c) and quantified (d, n = 7 independent biological samples). Sale bar = 20 μm.

e Rates of ROS production in A549 cybrid cells and the parental cells were measured using dihydroethidium (DHE), as the rate of increase in red fluorescence intensity over 80 min incubation with DHE.

f ROS production rates of A549 cybrid cells reported by DHE (n = 9 independent biological samples; p = 0.0003) and MitoSOX (n = 3 independent biological samples; p = 0.0026).

Source data are provided as a Source Data file. All data are represented as mean ± S.D. and were analysed by one-way ANOVA with Tukey's multiple comparisons test for fibroblasts and by unpaired t test for cybrid cells (* p < 0.05, ** p < 0.01, *** p < 0.001, **** p < 0.0001).

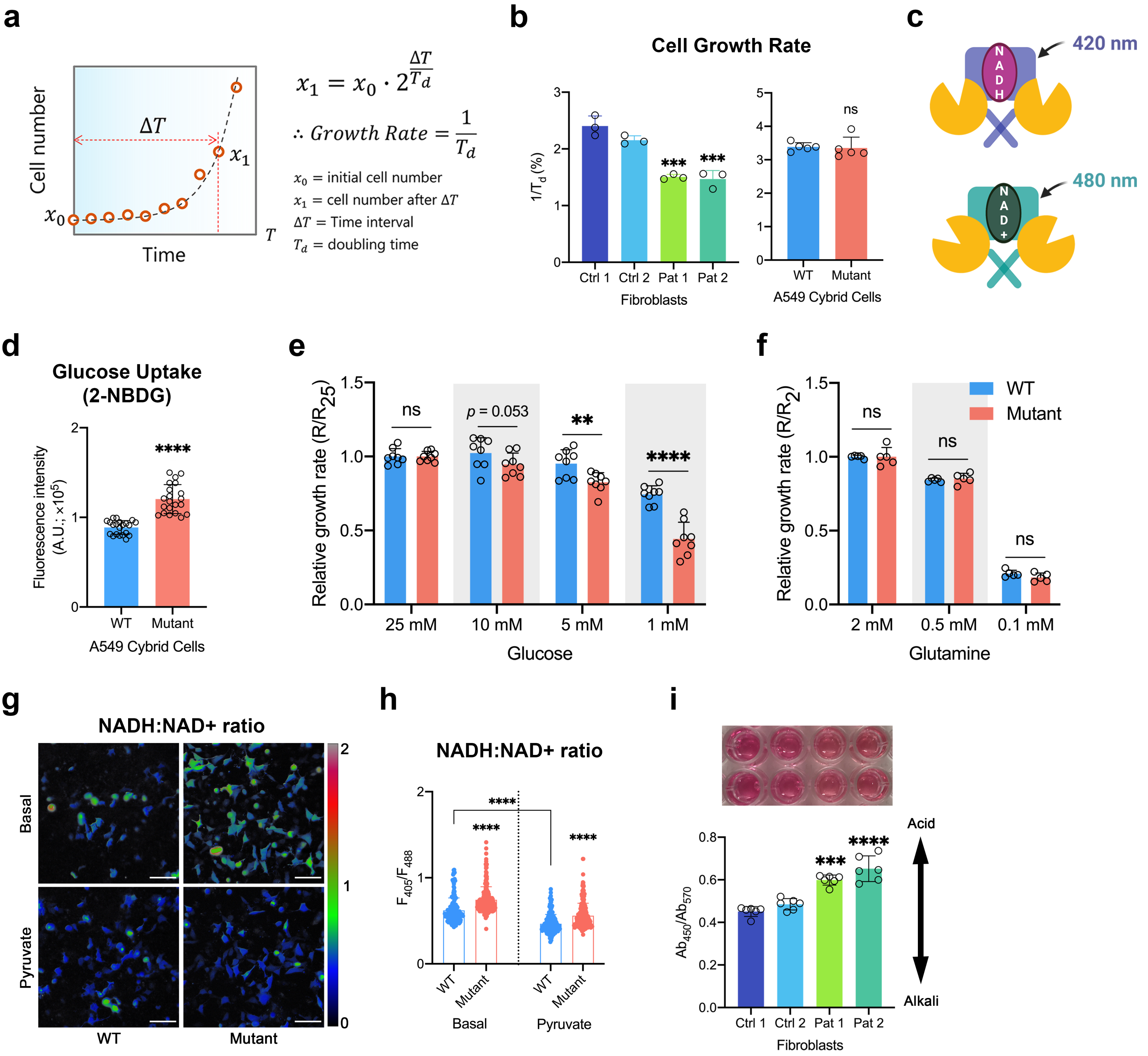

a
Cell number
Time
x_0
x_1
ΔT
T
$x_1 = x_0 \cdot 2^{\frac{\Delta T}{T_d}}$
$\therefore Growth\ Rate = \frac{1}{T_d}$
x_0 = initial cell number
x_1 = cell number after ΔT
ΔT = Time interval
T_d = doubling time
b
Cell Growth Rate
$1/T_d$ (%)
Ctrl 1
Ctrl 2
Pat 1
Pat 2
Fibroblasts
WT
Mutant
A549 Cybrid Cells
ns
***
c
420 nm
480 nm
NADH
NAD+
d
Glucose Uptake (2-NBDG)
Fluorescence intensity (A.U.; ×10^5)
****
WT
Mutant
A549 Cybrid Cells
e
Relative growth rate (R/R$_{25}$)
ns
p = 0.053
**
****
25 mM
10 mM
5 mM
1 mM
Glucose
f
Relative growth rate (R/R$_2$)
WT
Mutant
ns
2 mM
0.5 mM
0.1 mM
Glutamine
g
NADH:NAD+ ratio
Basal
Pyruvate
WT
Mutant
h
NADH:NAD+ ratio
F$_{405}$/F$_{488}$
****
WT
Mutant
Basal
Pyruvate
i
Ab$_{450}$/Ab$_{570}$
***
****
Acid
Alkali
Ctrl 1
Ctrl 2
Pat 1
Pat 2
Fibroblasts

Supplementary Fig. 2. The A549 cybrid cells also show increased glucose dependence.

a-b A scheme describing how growth rates were obtained by fitting growth curves with an exponential cell growth model (a). (b) Based on the model, growth rate of fibroblasts (n = 3 culture wells; $p < 0.0001$) and A549 cybrid cells cultured with regular medium were obtained (n = 5 culture wells, $p = 0.84$).

c Schematic depicting the ratiometric probe for NADH:NAD^+, SoNar.

d Glucose uptake in A549 cybrid cells was measured by 2-NBDG, showing a significantly increased rate of glucose uptake in the cybrid cells compared to A549 controls (n = 21 culture wells; $p < 0.0001$).

e-f Cell growth rates of A549 cybrid cells were measured under a range of different nutrient conditions (normalised to the growth rate of each cell line in regular cell media), showing a decreased rate of growth of A549 cybrid cells compared to controls at glucose concentrations of 5 and 1 mM (**e**; n = 10 culture wells; $p = 0.0024$ in 5 mM and $p < 0.000001$ in 1 mM) but not at low glutamine concentrations (**f**, n = 6 culture wells).

g-h NADH:NAD^+ ratio of A549 cybrid cells under basal condition (n = 181 and 194 cells for WT and Mutant, respectively) and after addition of pyruvate (200 μM, 30 min; n = 205 and 227 cells for WT and Mutant, respectively; **g**) was measured by the probe and quantified (**h**; $p < 0.0001$). Scale bar = 100 μm.

i pH in the media from patient fibroblasts cultured for 2 days was measured based on the ratiometric property of the pH indicator, phenol red, in media and showing a lower pH in the media of patient fibroblasts than that of controls (n = 6 independent biological samples; $p < 0.0001$).

Source data are provided as a Source Data file. All data are represented as mean ± S.D. and were analysed by one-way ANOVA with Tukey's multiple comparisons test for fibroblasts and by unpaired t test for cybrid cells (* $p < 0.05$, ** $p < 0.01$, *** $p < 0.001$, **** $p < 0.0001$).

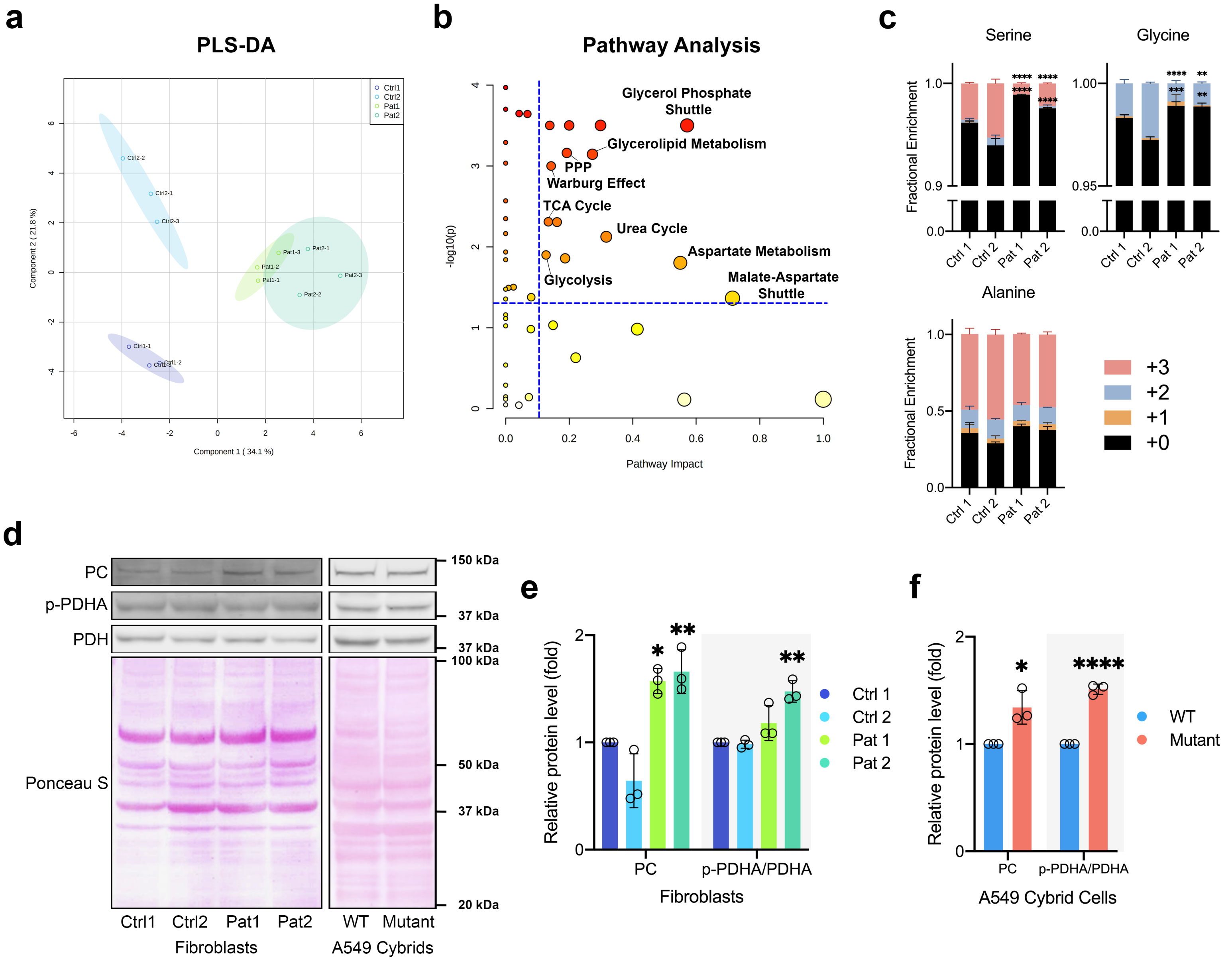
a
PLS-DA
Ctrl1
Ctrl2
Pat1
Pat2
Ctrl2-2
Ctrl2-1
Ctrl2-3
Pat1-3
Pat2-1
Pat1-2
Pat1-1
Pat2-3
Pat2-2
Ctrl1-1
Ctrl1-2
Component 2 (21.8 %)
Component 1 (34.1 %)
b
Pathway Analysis
Glycerol Phosphate Shuttle
Glycerolipid Metabolism
PPP
Warburg Effect
TCA Cycle
Urea Cycle
Aspartate Metabolism
Glycolysis
Malate-Aspartate Shuttle
-log10(p)
Pathway Impact
c
Serine
Glycine
Alanine
Fractional Enrichment
Ctrl 1
Ctrl 2
Pat 1
Pat 2
+3
+2
+1
+0
d
PC
p-PDHA
PDH
Ponceau S
150 kDa
37 kDa
37 kDa
100 kDa
50 kDa
37 kDa
20 kDa
Ctrl1
Ctrl2
Pat1
Pat2
WT
Mutant
Fibroblasts
A549 Cybrids
e
Relative protein level (fold)
PC
p-PDHA/PDHA
Fibroblasts
Ctrl 1
Ctrl 2
Pat 1
Pat 2
f
Relative protein level (fold)
PC
p-PDHA/PDHA
A549 Cybrid Cells
WT
Mutant

Supplementary Fig. 3. The metabolomic of fibroblasts carrying the m.3243A>G mutation are distinct from that of their matched controls.

a PLS-DA for the dataset of Fig. 3a showed that the metabolic profiles of patient fibroblasts were distinct from that of controls.

b The concentration of metabolites obtained by GC-MS was used to determine the enriched metabolic pathways in patient fibroblasts by MetaboAnalyst 5.0., suggesting that phospholipid biosynthesis, PPP, glycolysis, TCA cycle, etc. were enriched (n = 6 technical replicates).

c With an increase of serine and alanine concentration (Fig. 3a) in the patient cells, the incorporation of ^{13}C into serine ($p < 0.0001$ in m+0 and m+3) and glycine ($p < 0.0001$ in m+0 and m+2) were reduced, while alanine was not altered (n = 3 independent biological samples).

d-f Immunoblotting (**d**; n = 3 independent experiments for all cell lines) of the expression of pyruvate carboxylase and phosphorylation state of PDH showed an increase of pyruvate carboxylase expression and increased phosphorylation of PDH (S293) in patient fibroblasts (**e**; $p = 0.0003$ in PC and $p = 0.0009$ in p-PDH) and A549 cybrid cells (**f**; $p = 0.0190$ in PC and $p < 0.0001$ in p-PDH).

Source data are provided as a Source Data file. All data, except Supplementary Figs. 3a and 3b, are represented as mean ± S.D. and were analysed by one-way ANOVA with Tukey's multiple comparisons test for fibroblasts and by unpaired t test for cybrid cells (* $p < 0.05$, ** $p < 0.01$, *** $p < 0.001$, **** $p < 0.0001$)

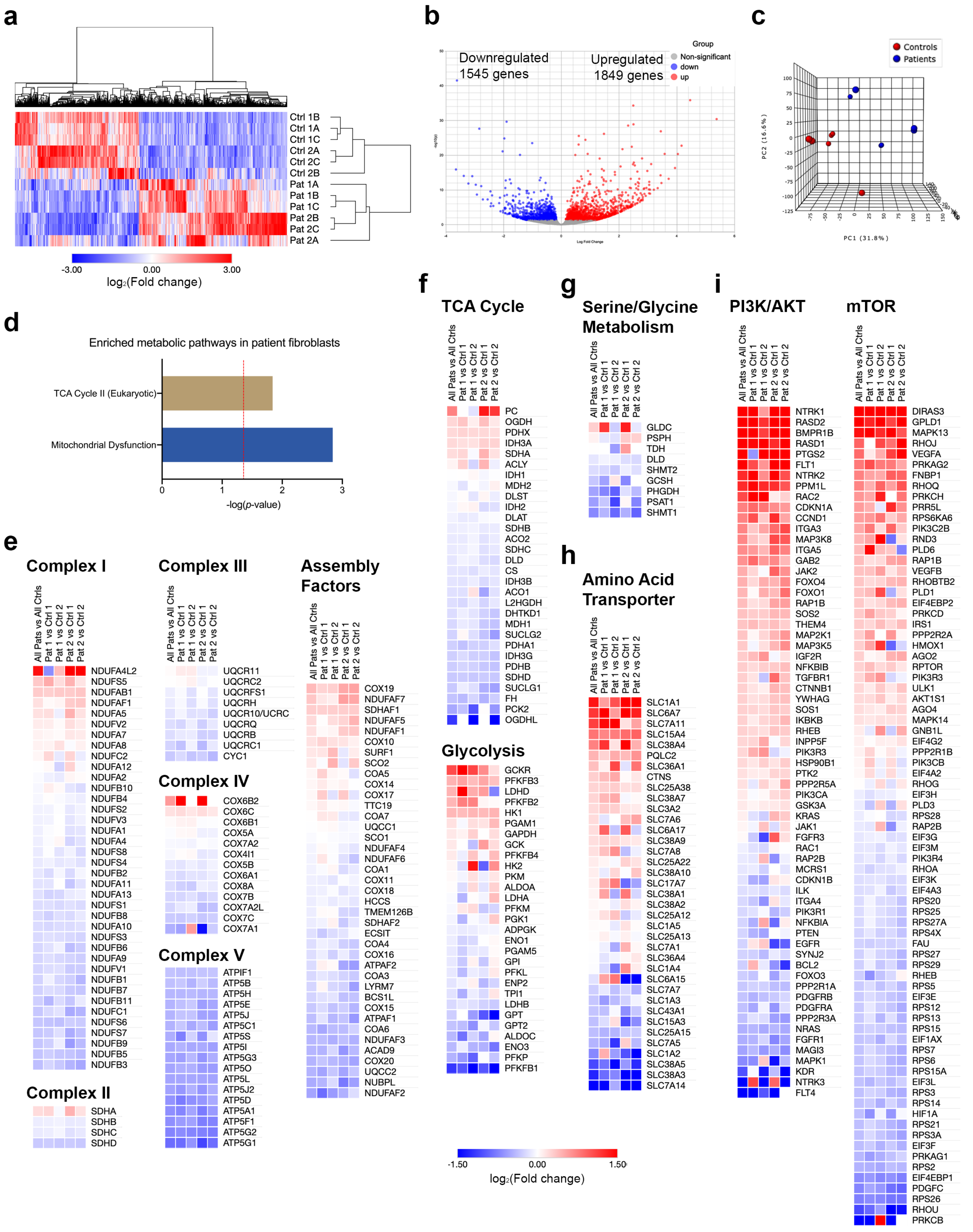
a
Ctrl 1B
Ctrl 1A
Ctrl 1C
Ctrl 2A
Ctrl 2C
Ctrl 2B
Pat 1A
Pat 1B
Pat 1C
Pat 2B
Pat 2C
Pat 2A
-3.00
0.00
3.00
log2(Fold change)
b
Downregulated 1545 genes
Upregulated 1849 genes
Group
Non-significant
down
up
c
Controls
Patients
PC2 (16.6%)
PC1 (31.8%)
d
Enriched metabolic pathways in patient fibroblasts
TCA Cycle II (Eukaryotic)
Mitochondrial Dysfunction
0
1
2
3
-log(p-value)
e
Complex I
All Pats vs All Ctrls
Pat 1 vs Ctrl 1
Pat 1 vs Ctrl 2
Pat 2 vs Ctrl 1
Pat 2 vs Ctrl 2
NDUFA4L2
NDUFS5
NDUFAB1
NDUFAF1
NDUFA5
NDUFV2
NDUFA7
NDUFA8
NDUFC2
NDUFA12
NDUFA2
NDUFB10
NDUFB4
NDUFS2
NDUFV3
NDUFA1
NDUFA4
NDUFS8
NDUFS4
NDUFB2
NDUFA11
NDUFA13
NDUFS1
NDUFB8
NDUFA10
NDUFS3
NDUFB6
NDUFA9
NDUFV1
NDUFB1
NDUFB7
NDUFB11
NDUFC1
NDUFS6
NDUFS7
NDUFB9
NDUFB5
NDUFB3
Complex II
SDHA
SDHB
SDHC
SDHD
Complex III
UQCR11
UQCRC2
UQCRFS1
UQCRH
UQCR10/UCRC
UQCRQ
UQCRB
UQCRC1
CYC1
Complex IV
COX6B2
COX6C
COX6B1
COX5A
COX7A2
COX4I1
COX5B
COX6A1
COX8A
COX7B
COX7A2L
COX7C
COX7A1
Complex V
ATPIF1
ATP5B
ATP5H
ATP5E
ATP5J
ATP5C1
ATP5S
ATP5I
ATP5G3
ATP5O
ATP5L
ATP5J2
ATP5D
ATP5A1
ATP5F1
ATP5G2
ATP5G1
Assembly Factors
COX19
NDUFAF7
SDHAF1
NDUFAF5
NDUFAF1
COX10
SURF1
SCO2
COA5
COX14
COX17
TTC19
COA7
UQCC1
SCO1
NDUFAF4
NDUFAF6
COA1
COX11
COX18
HCCS
TMEM126B
SDHAF2
ECSIT
COA4
COX16
ATPAF2
COA3
LYRM7
BCS1L
COX15
ATPAF1
COA6
NDUFAF3
ACAD9
COX20
UQCC2
NUBPL
NDUFAF2
f
TCA Cycle
PC
OGDH
PDHX
IDH3A
SDHA
ACLY
IDH1
MDH2
DLST
IDH2
DLAT
SDHB
ACO2
SDHC
DLD
CS
IDH3B
ACO1
L2HGDH
DHTKD1
MDH1
SUCLG2
PDHA1
IDH3G
PDHB
SDHD
SUCLG1
FH
PCK2
OGDHL
Glycolysis
GCKR
PFKFB3
LDHD
PFKFB2
HK1
PGAM1
GAPDH
GCK
PFKFB4
HK2
PKM
ALDOA
LDHA
PFKM
PGK1
ADPGK
ENO1
PGAM5
GPI
PFKL
ENP2
TPI1
LDHB
GPT
GPT2
ALDOC
ENO3
PFKP
PFKFB1
g
Serine/Glycine Metabolism
GLDC
PSPH
TDH
DLD
SHMT2
GCSH
PHGDH
PSAT1
SHMT1
h
Amino Acid Transporter
SLC1A1
SLC6A7
SLC7A11
SLC15A4
SLC38A4
PQLC2
SLC36A1
CTNS
SLC25A38
SLC38A7
SLC3A2
SLC7A6
SLC6A17
SLC38A9
SLC7A8
SLC25A22
SLC38A10
SLC17A7
SLC38A1
SLC38A2
SLC25A12
SLC1A5
SLC25A13
SLC7A1
SLC36A4
SLC1A4
SLC6A15
SLC7A7
SLC1A3
SLC43A1
SLC15A3
SLC25A15
SLC7A5
SLC1A2
SLC38A5
SLC38A3
SLC7A14
i
PI3K/AKT
NTRK1
RASD2
BMPR1B
RASD1
PTGS2
FLT1
NTRK2
PPM1L
RAC2
CDKN1A
CCND1
ITGA3
MAP3K8
ITGA5
GAB2
JAK2
FOXO4
FOXO1
RAP1B
SOS2
THEM4
MAP2K1
MAP3K5
IGF2R
NFKBIB
TGFBR1
CTNNB1
YWHAG
SOS1
IKBKB
RHEB
INPP5F
PIK3R3
HSP90B1
PTK2
PPP2R5A
PIK3CA
GSK3A
KRAS
JAK1
FGFR3
RAC1
RAP2B
MCRS1
CDKN1B
ILK
ITGA4
PIK3R1
NFKBIA
PTEN
EGFR
SYNJ2
BCL2
FOXO3
PPP2R1A
PDGFRB
PDGFRA
PPP2R3A
NRAS
FGFR1
MAGI3
MAPK1
KDR
NTRK3
FLT4
mTOR
DIRAS3
GPLD1
MAPK13
RHOJ
VEGFA
PRKAG2
FNBP1
RHOQ
PRKCH
PRR5L
RPS6KA6
PIK3C2B
RND3
PLD6
RAP1B
VEGFB
RHOBTB2
PLD1
EIF4EBP2
PRKCD
IRS1
PPP2R2A
HMOX1
AGO2
RPTOR
PIK3R3
ULK1
AKT1S1
AGO4
MAPK14
GNB1L
EIF4G2
PPP2R1B
PIK3CB
EIF4A2
RHOG
EIF3H
PLD3
RPS28
RAP2B
EIF3G
EIF3M
PIK3R4
RHOA
EIF3K
EIF4A3
RPS20
RPS25
RPS27A
RPS4X
FAU
RPS27
RPS29
RHEB
RPS5
EIF3E
RPS12
RPS13
RPS15
EIF1AX
RPS7
RPS6
RPS15A
EIF3L
RPS3
RPS14
HIF1A
RPS21
RPS3A
EIF3F
PRKAG1
RPS2
EIF4EBP1
PDGFC
RPS26
RHOU
PRKCB
-1.50
0.00
1.50
log2(Fold change)

Supplementary Fig. 4. Analysis for RNA-seq of patient fibroblasts with other supporting results and the enriched metabolic pathways concordant with the metabolic phenotype of patient fibroblasts.

a-c RNA-seq showed by a heatmap (**a**), a volcano plot (**b**) and 3D-PCA (**c**). Gene expression in the mutant cells was significantly distinct from both of the controls: 3394 genes were differentially expressed (FDR < 0.05, with 1849 up-regulated and 1545 down-regulated).

d Analysis of RNA-seq data from the patient fibroblasts by QIAGEN Ingenuity Pathway Analysis (IPA) showed enriched metabolic pathways ($p < 0.05$) that matches the findings shown in Figs. 1, 2 and 3.

e Analysis of the RNA seq data showing a general decrease in mRNA expression of OxPhos-related genes in patient fibroblasts.

f The mRNA expression of TCA cycle and glycolysis genes in patient fibroblasts confirmed our findings in Figs. 2 and 3, such as increased pyruvate carboxylase and HK1 expression.

g-h Analysis of the RNA-seq data showing the mRNA expression associated with amino acid metabolism, including serine/glycine metabolism (**g**) and amino acid transporters (**h**).

i Detailed analysis of the mRNA expression of multiple genes involved in the PI3K-Akt and mTOR pathways in patient fibroblasts, showing consistent differences in a wide array of genes involved in these pathways.

Source data are provided as a Source Data file.

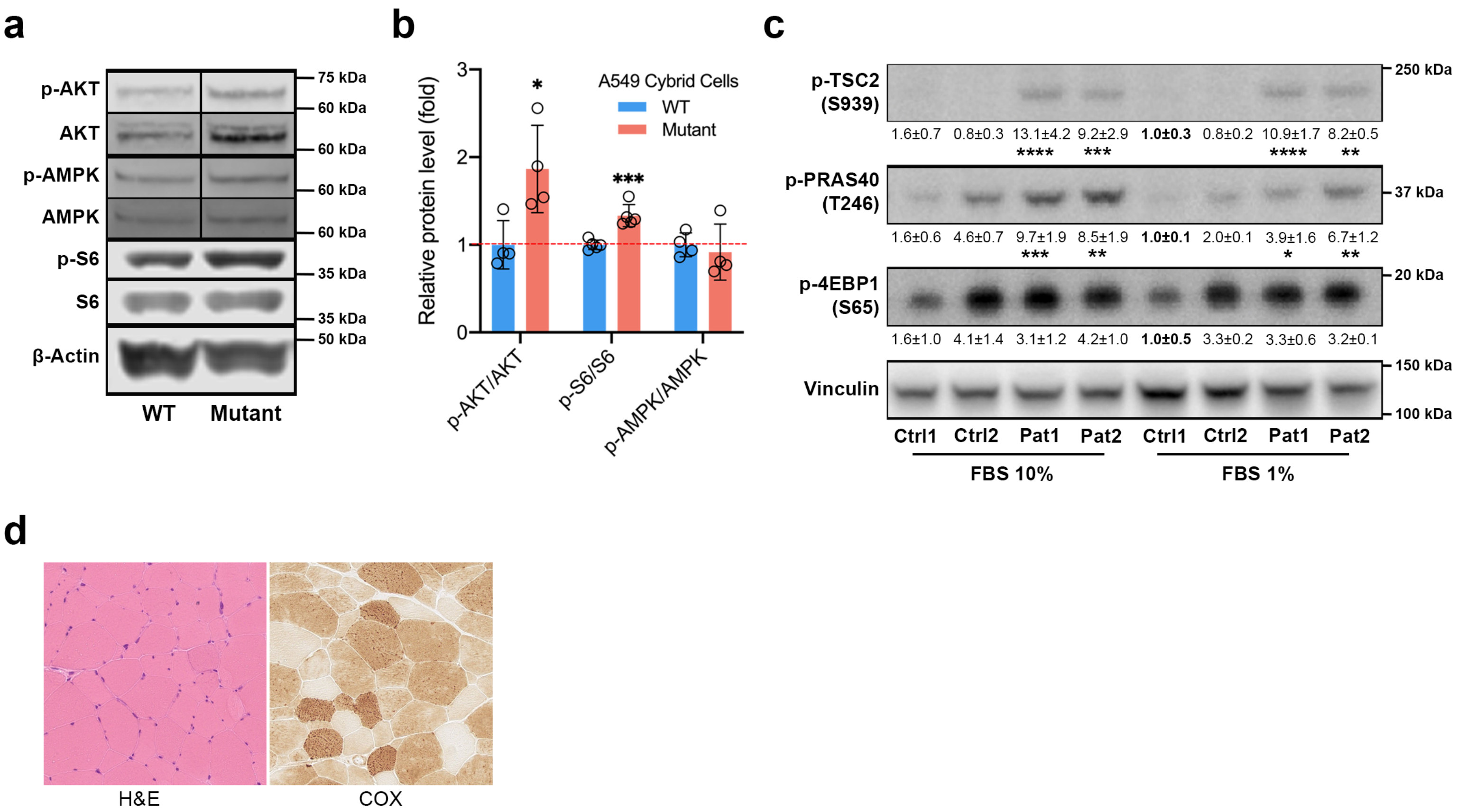
a
p-AKT
AKT
p-AMPK
AMPK
p-S6
S6
β-Actin
75 kDa
60 kDa
60 kDa
60 kDa
60 kDa
35 kDa
35 kDa
50 kDa
WT
Mutant
b
Relative protein level (fold)
A549 Cybrid Cells
WT
Mutant
0
1
2
3
*
***
p-AKT/AKT
p-S6/S6
p-AMPK/AMPK
c
p-TSC2 (S939)
1.6±0.7 0.8±0.3 13.1±4.2 9.2±2.9 1.0±0.3 0.8±0.2 10.9±1.7 8.2±0.5
**** *** **** **
p-PRAS40 (T246)
1.6±0.6 4.6±0.7 9.7±1.9 8.5±1.9 1.0±0.1 2.0±0.1 3.9±1.6 6.7±1.2
*** ** * **
p-4EBP1 (S65)
1.6±1.0 4.1±1.4 3.1±1.2 4.2±1.0 1.0±0.5 3.3±0.2 3.3±0.6 3.2±0.1
Vinculin
250 kDa
37 kDa
20 kDa
150 kDa
100 kDa
Ctrl1 Ctrl2 Pat1 Pat2 Ctrl1 Ctrl2 Pat1 Pat2
FBS 10%
FBS 1%
d
H&E
COX

Supplementary Fig. 5. Other supporting results from patient fibroblasts and A549 cybrid cells for the activity of the PI3K-Akt-mTORC1 axis.

a-b Immunoblotting of p-Akt (S473)/Akt, p-S6 (S235/236)/S6 and p-AMPK (T172)/AMPK in A549 cybrid cells (**a**). (**b**) Quantitation shows increased phosphorylation of Akt (n = 5; p = 0.0229) and S6 (n = 5; p = 0.0006) but not of AMPK (n = 4 independent experiments; p = 0.6467), consistent with the results of fibroblasts.

c Immunoblotting of Akt substrates, p-TSC2 (S939) and p-PRAS40 (T246), and of another mTORC1 substrate, p-4EBP1 (S65), in patient fibroblasts grown in the presence of 10% or 1% FBS media shows increased phosphorylation (n = 3 independent experiments).

d H&E and COX staining of the corresponding patient biopsy in Fig. 4d. Scale bar = 100 μm.

Source data are provided as a Source Data file. All data are represented as mean ± S.D. and were analysed by one-way ANOVA with Tukey's multiple comparisons test for fibroblasts and by unpaired t test for cybrid cells (* $p < 0.05$, ** $p < 0.01$, *** $p < 0.001$, **** $p < 0.0001$).

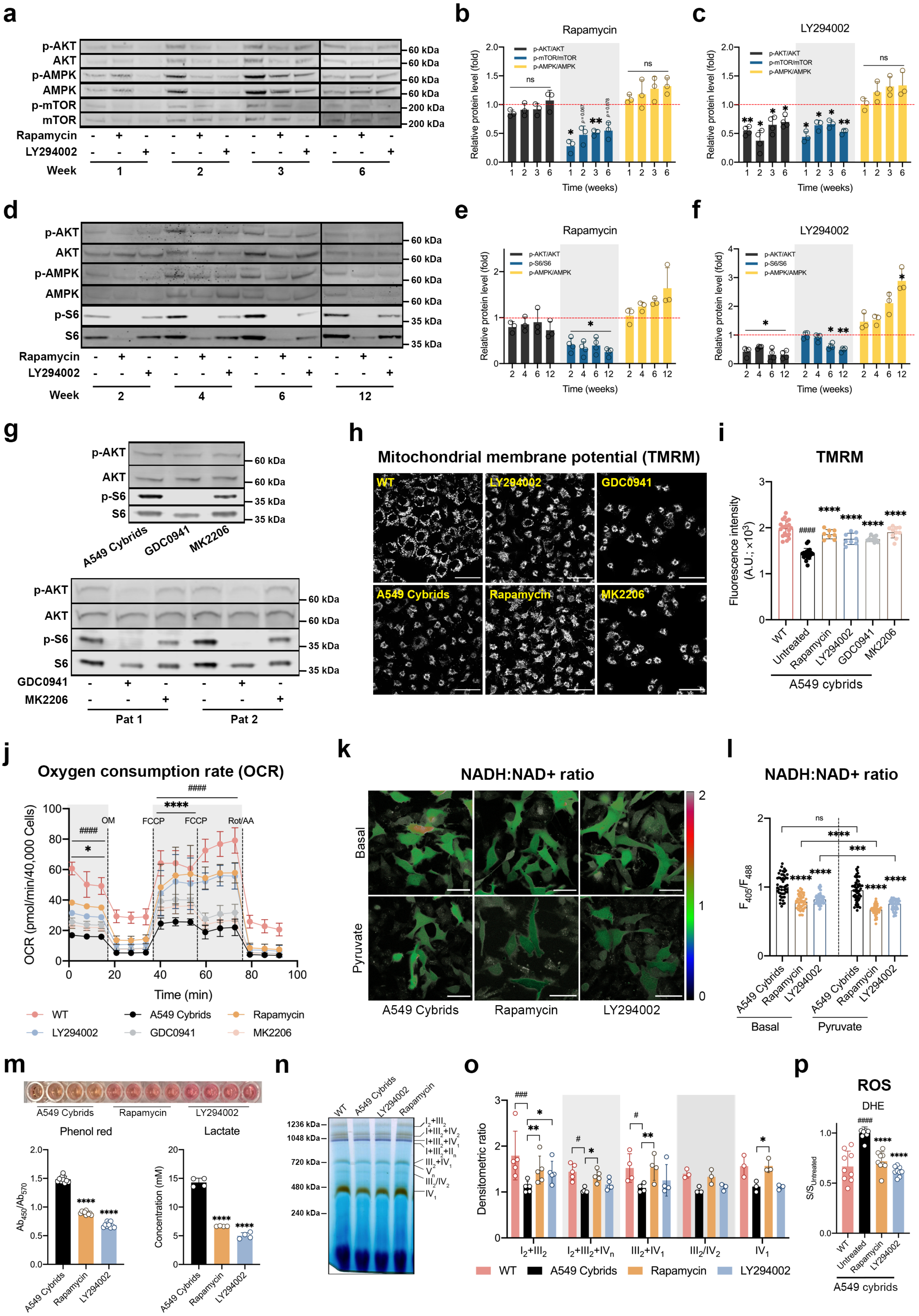
a
p-AKT
AKT
p-AMPK
AMPK
p-mTOR
mTOR
60 kDa
200 kDa
Rapamycin
LY294002
Week
1
2
3
6
b
Rapamycin
Relative protein level (fold)
p-AKT/AKT
p-mTOR/mTOR
p-AMPK/AMPK
ns
Time (weeks)
c
LY294002
d
p-S6
S6
35 kDa
12
e
p-S6/S6
f
g
A549 Cybrids
GDC0941
MK2206
Pat 1
Pat 2
h
Mitochondrial membrane potential (TMRM)
WT
i
TMRM
Fluorescence intensity (A.U.; ×10³)
Untreated
A549 cybrids
j
Oxygen consumption rate (OCR)
OCR (pmol/min/40,000 Cells)
OM
FCCP
Rot/AA
Time (min)
k
NADH:NAD+ ratio
Basal
Pyruvate
l
NADH:NAD+ ratio
F405/F488
m
Phenol red
Lactate
Ab450/Ab570
Concentration (mM)
n
1236 kDa
1048 kDa
720 kDa
480 kDa
240 kDa
I2+III2
I+III2+IV2
I+III2+IV1
I+III2+IIn
III2+IV1
Vn
III2/IV2
IV1
o
Densitometric ratio
I2+III2
I2+III2+IVn
III2+IV1
III2/IV2
IV1
p
ROS
DHE
S/SUntreated

Supplementary Fig. 6. Inhibitors of the PI3K-Akt-mTORC1 axis, LY294002 and Rapamycin, also reduced mutant load and partially rescued mitochondrial function in A549 cybrid cells.

a-c Immunoblotting of the phosphoproteins (p-Akt/Akt, p-mTOR/ mTOR and p-AMPK/AMPK; **a**) in A549 cybrid cells treated with RP (**b**; $p < 0.0001$) or LY (**c**; $p < 0.0001$) over 6 weeks demonstrate the effective inhibition of Akt or mTORC1 (n = 3 independent experiments).

d-f Immunoblotting of the phosphoproteins (p-Akt/Akt, p-S6/S6 and p-AMPK/AMPK; **d**) in patient 1 fibroblasts treated with RP (**e**; $p < 0.0001$) or LY (**f**; $p < 0.0001$) over 12 weeks demonstrated the effective inhibition of Akt or mTORC1 in the drug-treated cells (n = 3 independent experiments).

g Immunoblotting of the phosphoproteins (p-Akt/Akt and p-S6/S6) in A549 cybrid cells and in patient fibroblasts treated with GDC or MK over 6 weeks demonstrated the effective inhibition of Akt or mTORC1 and upregulated autophagic flux in the drug-treated cells (n = 3 independent experiments).

h-i The mitochondrial membrane potential of A549 cybrid cells (**h**, TMRM 25 nM) significantly increased after exposure to LY, GDC, MK or RP for 6 weeks to the level that only slightly lower than which of WT (**i**, n = 8 independent biological samples; $p < 0.0001$). Scale bar = 25 μm.

j Cell respiratory capacity of A549 cybrid cells treated with LY or RP for 6 weeks was measured using the Seahorse XFe96 extracellular flux analyser and showed a major increase in basal and maximum OCR after treatment (n = 18 culture wells; $p < 0.0001$).

k-l Cytosolic NADH:NAD^+ ratio of A549 cybrid cells transfected with the genetically encoded probe SoNar and treated with LY or RP for 6 weeks was measured (**k**) and quantified (**l**) under basal condition (n = 51, 51 and 64 cells for cybrid cells, RP and LY, respectively) and following exposure to pyruvate (200 μm, 30 min; n = 55, 50 and 50 cells for cybrid cells, RP and LY, respectively; $p < 0.0001$).

m The absorption ratio of phenol red was used to measure the pH of the growth media (n = 10 culture wells; $p < 0.0001$) and the kit of the CuBiAn instrument was used to measure lactate production (n = 4 culture wells; $p < 0.0001$). Both acidification of the medium and lactate secretion (48h incubation) of A549 cybrid cells treated with LY or RP for 6 weeks were reduced.

n-o BNGE were used to measure supercomplex assembly and In Gel activity of A549 cybrid cells after 6 weeks of treatment with LY or RP (**n**) and quantified (**o**, n = 3 independent experiments; $p < 0.0001$).

p Rates of ROS production of A549 cybrid cells treated with LY or RP for 6 weeks were reduced to the level that no longer significantly different from WT cells (n = 9 culture wells; $p < 0.0001$).

Source data are provided as a Source Data file. All data represented as mean ± S.D. and were analysed by one/two-way ANOVA with Tukey's multiple comparisons test (* $p < 0.05$, ** $p < 0.01$, *** $p < 0.001$,

**** $p < 0.0001$, vs Patient/Mutant controls; # $p < 0.05$, ## $p < 0.01$, ### $p < 0.001$, #### $p < 0.0001$, vs WT controls).

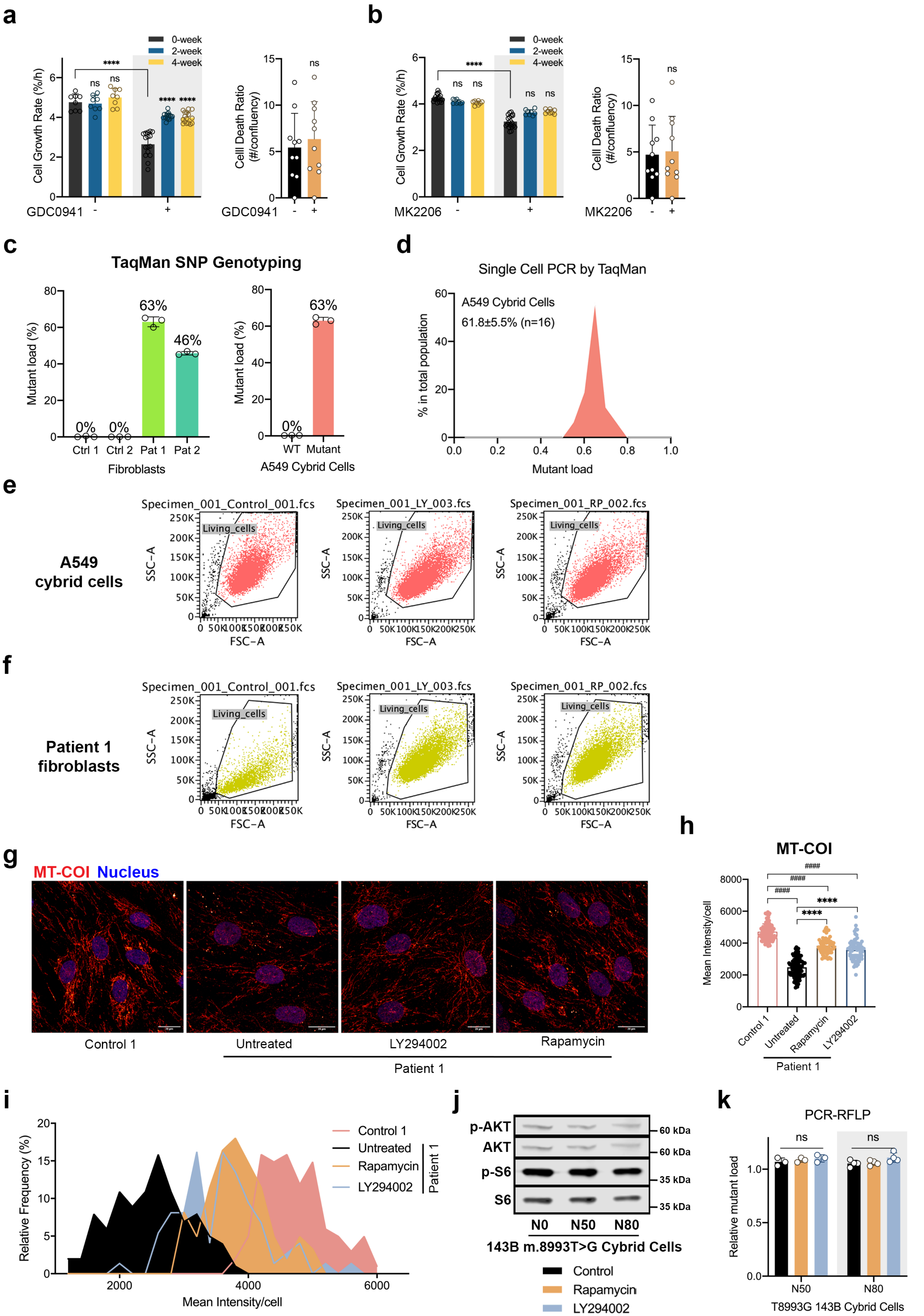
a
Cell Growth Rate (%/h)
0-week
2-week
4-week
GDC0941
Cell Death Ratio (#/confluency)
b
MK2206
c
TaqMan SNP Genotyping
Mutant load (%)
Ctrl 1
Ctrl 2
Pat 1
Pat 2
Fibroblasts
WT
Mutant
A549 Cybrid Cells
d
Single Cell PCR by TaqMan
A549 Cybrid Cells
61.8±5.5% (n=16)
% in total population
Mutant load
e
A549 cybrid cells
Specimen_001_Control_001.fcs
Specimen_001_LY_003.fcs
Specimen_001_RP_002.fcs
Living_cells
SSC-A
FSC-A
f
Patient 1 fibroblasts
g
MT-COI Nucleus
Control 1
Untreated
LY294002
Rapamycin
Patient 1
h
MT-COI
Mean Intensity/cell
i
Relative Frequency (%)
Mean Intensity/cell
j
p-AKT
AKT
p-S6
S6
60 kDa
35 kDa
N0
N50
N80
143B m.8993T>G Cybrid Cells
Control
Rapamycin
LY294002
k
PCR-RFLP
Relative mutant load
T8993G 143B Cybrid Cells

Supplementary Fig. 7. TaqMan SNP genotyping can be used at single cell resolution to measure the distribution of mutant mtDNA burden in a cell population.

a-b Cell growth and cell death of A549 cybrid cells were measured before and after treatment with GDC (**a**) or MK (**b**) for 4 weeks (n = 8 culture wells for all groups; $p < 0.0001$). The drugs slight inhibited cell growth but had no specific effect on the cell death of mutant cells.

c-d TaqMan SNP genotyping was validated by measuring the mutation load in a whole population of cells (**c**, n = 3 independent biological samples) and then to establish the range of mutant load at the level of single cells in the A549 cybrid cells (**d**).

e-f Sorting strategies for A549 cybrid cells (**e**) and patient 1 fibroblasts (**f**) for single-cell PCR.

g-i Immunofluorescence staining for MT-COI (**g** and quantified in **h**; $p < 0.0001$) showed that the expression of MT-COI in patient 1 fibroblasts (n = 101 cells) was significantly lower than that of control 1 (n = 101 cells), while LY (n = 74 of cells) or RP (n = 61 of cells) treatments restored its expression. The histograms (**i**) display the heterogeneous distribution of intensities measured at the single cell level in control 1 and patient 1 fibroblasts.

j-k Immunoblotting of the phosphoproteins (p-Akt/Akt and p-S6/S6) in 143B cybrid cells bearing the m.8993T>G mutation showed no difference in protein phosphorylation among cells with 0% (N0), 50% (N50) and 80% (N80) mutant loads (**j**, n = 3 independent experiments). PCR-RFLP was applied to measure the change in mutant load in 143B cybrid cells carrying the m.8993T>G mutation treated with LY or RP for 8 weeks, showing no significant effect on the mutant load of the m.8993T>G (**k**, n = 3 independent biological samples).

Source data are provided as a Source Data file. All data are represented as mean ± S.D. and were analysed by one/two-way ANOVA with Tukey's multiple comparisons test (* $p < 0.05$, ** $p < 0.01$, *** $p < 0.001$, **** $p < 0.0001$).

Supplementary Table 1. Upstream analysis for Kinases in patient fibroblasts by IPA.

Kinase	Prediction	Z-score	*p*-value
CDKN1A	Activated	3.084	1.12E-08
IKBKB		3.244	0.000377
**AKT1**		2.47	0.00201
MAPK13		2.079	0.00245
TGFBR2		2.747	0.00255
PTK2		2.48	0.00302
CHUK		4.427	0.00929
STK11	Inhibited	-2.494	0.00000766
INSR		-2.032	0.000675
CCNK		-2.399	0.000866
AURKB		-2.772	0.005

Bold, genes of the PI3K-Akt-mTOR axis.

Supplementary Table 2. Upstream analysis using published RNA-seq dataset of patient biopsies by IPA.

Upstream Regulator	Molecule Type	*p*-value
TRIB3	Kinase	0.0000295
**MTOR**		0.00112
TGFBR2		0.0101
GNE		0.0155
**PIK3R1**		0.0159
TGFBR1		0.0166
CDK19		0.0199
Growth hormone	Group	0.000165
**Foxo**		0.000504
ADRB		0.00393
Insulin		0.00402
YAP/TAZ		0.0089
Pro-inflammatory Cytokine		0.0101
**PI3K (family)**		0.026
Rxr		0.0269
**ATF4**	Transcription Regulator	0.00048
MLX		0.000905
PPARGC1A		0.00115
EP300		0.0029
FOXO1		0.00299
MLXIPL		0.00326
MLXIP		0.00446
ZNF282		0.0089
MYRF		0.0104
HDAC5		0.0122
NKX2-3		0.0127
MYC		0.0131
STAT6		0.0149
ZNF100		0.0155
ZNF85		0.0155
ZNF254		0.0155
ZNF431		0.0155
ZNF43		0.0155
ZNF429		0.0155
HDAC10		0.0177
ZNF91		0.0177
**ATF5**		0.0221
PA2G4		0.0221
KAT5		0.0231
STAT5B		0.026
AJUBA		0.0265
**DDIT3**		0.0282
BHLHE41		0.0287
ARNT		0.0304
PIAS4		0.0308
THRAP3		0.033
PLAGL1		0.033
KLF11		0.0337
CREBZF		0.0352
CBX4		0.0352
TCF7L2		0.0374
MNT		0.0416
ACTN4		0.0416
CREB3L3		0.0438
NONO		0.0438
MED12		0.0459
SIN3B		0.0459
PBX3		0.048
HNF4A		0.048
MED1		0.0492

Bold, genes of the PI3K-Akt-mTOR axis or the ISR pathway.

Supplementary Table 3. Upstream analysis for transcription regulators in patient fibroblasts by IPA.

Transcription regulator	Prediction	Z-score	*p*-value
TP53	Activated	3.282	2.79E-21
NUPR1		4.066	5.29E-11
KDM5B		3.627	0.00000101
CTNNB1		2.96	0.00000321
NFKBIA		2.632	0.0000157
GLI1		2.971	0.0000256
TCF4		2.225	0.0000457
SMARCA4		3.609	0.000108
KLF4		2.946	0.000627
PTTG1		2.302	0.000791
TFEB		3.273	0.0008
CDKN2A		4.62	0.000838
TCF7L2		4.059	0.000841
TCF3		3.926	0.00118
HIF1A		2.117	0.00224
CREB1		3.164	0.00238
HDAC1		2.228	0.00248
RBCK1		2.236	0.00252
E2F6		2.53	0.00259
**ATF4**		2.495	0.00296
SMARCD3		2.813	0.005
HDAC2		2.468	0.00621
ERG		2.557	0.00988
EP300		2.298	0.00994
TBX2	Inhibited	-4.488	2.43E-08
MYC		-4.01	2.46E-07
MYCN		-5.406	0.00000442
GMNN		-2.746	0.0000098
SOX1		-3	0.0000215
HOXA9		-2.01	0.0000273
E2F3		-3.186	0.0000273
SOX3		-3	0.00012
RUNX3		-2.272	0.000213

Bold, genes of the ISR pathway.

Supplementary Table 4. Key source table.

Reagent or Resource	Source	Identifier
**Antibodies**		
Mouse Ab anti-OxPhos(human) cocktail (1:1000)	Invitrogen	Cat#45-8199
Rabbit mAb anti-SDHA (1:1000)	Abcam	Cat#ab137040
Mouse mAb anti-ATP5A [15H4C4] (1:1000)	Abcam	Cat#ab14748
Rabbit pAb anti-PC (1:1000)	Novus Biologicals	Cat#NBP1-49536
Rabbit pAb anti-phospho-PDHA(Ser293) (1:1000)	Millipore	Cat#AP1062
Mouse pAb anti-PDHA (1:1000)	Invitrogen	Cat#45-6600
Rabbit pAb anti-phospho-Akt(Ser473) (1:1000)	Cell Signaling Technology	Cat#9271
Rabbit pAb anti-Akt (1:3000)	Cell Signaling Technology	Cat#9272
Rabbit mAb anti-phospho-S6 ribosomal protein (Ser235/236) (1:3000 IB; 1:200 IHC)	Cell Signaling Technology	Cat#4858
Rabbit mAb anti-S6 ribosomal protein (1:3000 IB; 1:200 IHC)	Cell Signaling Technology	Cat#2217
Rabbit mAb anti-phospho-mTOR (Ser2448) [D9C2] (1:1000)	Cell Signaling Technology	Cat#5536
Rabbit mAb anti-mTOR [7C10] (1:3000)	Cell Signaling Technology	Cat#2983
Rabbit mAb anti-phospho-AMPKα(Thr172) [40H9] (1:1000)	Cell Signaling Technology	Cat#2535
Rabbit pAb anti-AMPKα (1:3000)	Cell Signaling Technology	Cat#2532
Mouse mAb anti-β-actin[C4] (1:10000)	Santa Cruz Biotechnology	Cat#sc-47778
Goat anti-Mouse IgG IRDye® 680RD	Li-COR Biosciences	Cat#926-68070
Goat anti-Rabbit IgG IRDye® 800CWV	Li-COR Biosciences	Cat#926-32211
Mouse mAb anti-Akt(pan)[40D4] (1:200)	Cell Signaling Technology	Cat#2920
Rabbit mAb anti-phospho-Akt(Ser473) (1:200)	Abcam	Cat#ab81283
Mouse mAb anti-MTCO1[1D6E1A8] (1:100)	Abcam	Cat#ab14705
**Biological Samples**		
Human muscle biopsies	Division of Neuropathology, UCL Queen Square Institute of Neurology	https://www.ucl.ac.uk/ion/clinical-divisions/division-neuropathology
**Chemicals, Peptides, and Recombinant Proteins**		
Rapamycin mTORC1 inhibitor	Cayman Chemical	Cat#13346
LY294002 PI3K inhibitor	Cayman Chemical	Cat#70920
GDC0941 PI3K inhibitor	Cayman Chemical	Cat#11600
MK2206 AKT inhibitor	Cayman Chemical	Cat#11593
Tetramethylrhodamine methyl ester (TMRM)	Invitrogen	Cat#T668
Dihydroethidium (DHE)	Invitrogen	Cat#D11347
MitoSOX	Invitrogen	Cat#M36008
2-NBDG	Invitrogen	Cat#N13195
SYTOX Green Nucleic Acid Stain	Invitrogen	Cat#S7020
^{13}C-glucose	Goss Scientific	Cat#CLM-1396-1
**Critical Commercial Assays**		
Seahorse XF Cell Mito Stress Test Kit	Agilent	Cat#103015-100
Seahorse XFe96 FluxPak	Agilent	Cat#102416-100
Glucose Assay (384 tests)	OPTOCELL technology	Cat#200106
Lactate Assay (348 tests)	OPTOCELL technology	Cat#200115
Lipofectamine™ 3000 Transfection Reagent	Invitrogen	Cat#L3000001
Human Dermal Fibroblasts Nucleofector Kit	Lonza	Cat#VPD-1001
The Custom TaqMan SNP Genotyping Kit	Applied Biosystems	Cat#4332073
TaqPath ProAmp Master Mix	Applied Biosystems	Cat#A30866

**Experimental Models: Cell Lines**		
A549 cybrid cells	Ian Holt (MRC National Institute for Medical Research, London)	N/A
143B cybrid cells	Michael Minczuk (MRC Mitochondrial Biology Unit, Cambridge, UK)	N/A
Human fibroblasts (controls and patients)	MRC CNMD Biobank London	https://www.ucl.ac.uk/child-health/mrc-cnmd-biobank-london
**Oligonucleotides**		
Primers for the m.3243A>G PCR-RFLP:<br>Forward: GCCTACTTCACAAAGCGCCTTC;<br>Reverse: AGAAGAGCGATGGTGAGAGCTAAG	Nigou et al., 1998	Sigma-Aldrich
Primers for the m.3243A>G ARMS-qPCR:<br>Forward (3243A): CAGGGTTTGTTAAGATGGCAtA;<br>Forward (3243G): CAGGGTTTGTTAAGATGGCAtG;<br>Reverse: TGGCCATGGGTATGTTGTTA	Wang et al., 2011	Sigma-Aldrich
Primers for the m.8993T>G PCR-RFLP:<br>Forward: CCGACTAATCACCACCCAAC;<br>Reverse: TGTCGTGCAGGTAGAGGCTT	White et al., 2005	Sigma-Aldrich
**Recombinant DNA**		
SoNar (NADH:NAD$^+$ ratio reporter)	Zhao et al., 2015	N/A
**Software and Algorithms**		
Seahorse Wave Desktop 2.6 software	Agilent	https://www.agilent.com/en/products/cell-analysis/cell-analysis-software/data-analysis/wave-desktop-2-6
Fiji	Schindelin et al., 2012	https://imagej.net/Fiji
MetaboAnalyst 5.0	Chong et al., 2019	https://www.metaboanalyst.ca/MetaboAnalyst/home.xhtml
Ingenuity Pathway Analysis	Qiagen	Cat#830018
Morpheus	The Broad Institute	https://software.broadinstitute.org/morpheus
NetworkAnalyst 3.0	Xia et al., 2015	https://www.networkanalyst.ca/NetworkAnalyst/home.xhtml
Custom TaqMan® Assay design tool	Thermo Fisher	https://www.thermofisher.com/order/custom-genomic-products/tools/genotyping/
**Other**		
DMEM, no glucose, no glutamine, no phenol red	Gibco	Cat#A1443001
DMEM, no glucose	Gibco	Cat#11966025
DMEM, high glucose, no glutamine	Gibco	Cat#11960044
Dialyzed Fetal Bovine Serum	Sigma-Aldrich	Cat#F0392
