# Supplementary material for: Constitutive activation of the PI3K-Akt-mTORC1 pathway sustains the m.3243 A > G mtDNA mutation
Source: Nat Commun. 2021 Nov 4;12:6409. doi: 10.1038/s41467-021-26746-2 (PMC8568893; doi:10.1038/s41467-021-26746-2)
Supplement: Supplementary file 3 — Reporting Summary [file 41467_2021_26746_MOESM3_ESM.pdf]

nature research

Corresponding author(s): Michael R Duchen

Last updated by author(s): Oct 10, 2021

Reporting Summary

Nature Research wishes to improve the reproducibility of the work that we publish. This form provides structure for consistency and transparency in reporting. For further information on Nature Research policies, see our Editorial Policies and the Editorial Policy Checklist.

Statistics

For all statistical analyses, confirm that the following items are present in the figure legend, table legend, main text, or Methods section.

n/a	Confirmed	
☐	☒	The exact sample size (*n*) for each experimental group/condition, given as a discrete number and unit of measurement
☐	☒	A statement on whether measurements were taken from distinct samples or whether the same sample was measured repeatedly
☐	☒	The statistical test(s) used AND whether they are one- or two-sided<br>*Only common tests should be described solely by name; describe more complex techniques in the Methods section.*
☒	☐	A description of all covariates tested
☒	☐	A description of any assumptions or corrections, such as tests of normality and adjustment for multiple comparisons
☐	☒	A full description of the statistical parameters including central tendency (e.g. means) or other basic estimates (e.g. regression coefficient) AND variation (e.g. standard deviation) or associated estimates of uncertainty (e.g. confidence intervals)
☐	☒	For null hypothesis testing, the test statistic (e.g. *F*, *t*, *r*) with confidence intervals, effect sizes, degrees of freedom and *P* value noted<br>*Give P values as exact values whenever suitable.*
☒	☐	For Bayesian analysis, information on the choice of priors and Markov chain Monte Carlo settings
☒	☐	For hierarchical and complex designs, identification of the appropriate level for tests and full reporting of outcomes
☒	☐	Estimates of effect sizes (e.g. Cohen's *d*, Pearson's *r*), indicating how they were calculated

Our web collection on statistics for biologists contains articles on many of the points above.

Software and code

Policy information about availability of computer code

Data collection	CFX Maestro 2 (Bio-Rad) for qPCR, Seahorse Wave Desktop 2.6 (Agilent) for the Seahorse assay, Image Studio 5.0 (LI-COR) for immunoblotting, Zen Black (Zeiss) for confocal imaging, Incucyte Zoom (EssenBioScience) for live cell imaging, MassHunter Quantitative Analysis (B.06.00 SP01, Agilent Technologies) for GC-MS, and the SARTools R package for RNA-seq.
Data analysis	CFX Maestro 2 (Bio-Rad) for qPCR, Seahorse Wave Desktop 2.6 (Agilent) for the Seahorse assay, Image Studio 5.0 (LI-COR) for immunoblotting, Fiji for confocal imaging, Incucyte Zoom (EssenBioScience) for live cell imaging, Microsoft Excel 2019 for cell growth model fitting, MassHunter Quantitative Analysis (B.06.00 SP01, Agilent Technologies) and MetaboAnalyst 5.0 for GC-MS, Ingenuity Pathway Analysis, BD FACSdiva 7 for single-cell sorting, NetworkAnalyst 3.0 and Morpheus for RNA-seq, and GraphPad Prism 8 for all statistical analyses.

For manuscripts utilizing custom algorithms or software that are central to the research but not yet described in published literature, software must be made available to editors and reviewers. We strongly encourage code deposition in a community repository (e.g. GitHub). See the Nature Research guidelines for submitting code & software for further information.

Data

Policy information about availability of data

All manuscripts must include a data availability statement. This statement should provide the following information, where applicable:

- Accession codes, unique identifiers, or web links for publicly available datasets
- A list of figures that have associated raw data
- A description of any restrictions on data availability

Raw and processed RNA-seq data are accessible at the Gene Expression Omnibus under accession GSE175477.

Figs. 1-6 and Figs. S1-7 have associated raw data data, including uncropped images of immunoblotting, are available in the Source Data.

Field-specific reporting

Please select the one below that is the best fit for your research. If you are not sure, read the appropriate sections before making your selection.

☒ Life sciences ☐ Behavioural & social sciences ☐ Ecological, evolutionary & environmental sciences

For a reference copy of the document with all sections, see nature.com/documents/nr-reporting-summary-flat.pdf

Life sciences study design

All studies must disclose on these points even when the disclosure is negative.

Sample size	No sample size calculation was performed, sample sizes were chosen based on previous experience and on what is common practice in the field. For imaging, cell numbers are usually about 100 ; for single cell PCR analysis, cell numbers are usually about 500 and for immunoblotting and other assays, sample sizes are usually more than 3.
Data exclusions	No data were excluded.
Replication	Except RNA-seq, metabolomics, and single-cell PCR, all the other experiments were reproduced independently at least three times. ForRNA-seq and metabolomics, each cell line had 3 technical replications. For single cell PCR analysis, cell numbers for each cell line are about 500.
Randomization	For metabolomics analysis, the sample order was randomised during the data acquisition. For imaging, controls were measured first and then patient/mutant cell lines all at the same day in each replication. For other experiments, measurements were conducted with control and mutant cell lines together.
Blinding	This is not relevant to our study, since no subjective rating of data was involved and the reported data was based on quantitative measurements. Also, for a experiment, the person who conducted it and analyse data is usually the same one, so that blinding is impossible.

Reporting for specific materials, systems and methods

We require information from authors about some types of materials, experimental systems and methods used in many studies. Here, indicate whether each material, system or method listed is relevant to your study. If you are not sure if a list item applies to your research, read the appropriate section before selecting a response.

Materials & experimental systems

n/a	Involved in the study	
☐	☒	Antibodies
☐	☒	Eukaryotic cell lines
☒	☐	Palaeontology and archaeology
☒	☐	Animals and other organisms
☐	☒	Human research participants
☒	☐	Clinical data
☒	☐	Dual use research of concern

Methods

n/a	Involved in the study	
☒	☐	ChIP-seq
☐	☒	Flow cytometry
☒	☐	MRI-based neuroimaging

Antibodies

Antibodies used

All primary antibodies have anti-human protein reactivity.
Mouse Anti-OxPhos antibody cocktail (1:1000, Invitrogen #45-8199),
Rabbit anti-SDHA [EPR9043(B)](1:1000, Abcam #ab137040),
Mouse anti-ATP5A [15H4C4] (1:1000, Abcam #ab14748),
Rabbit anti-PC (1:1000, Novus Biologicals #NBP1-49536),
Rabbit anti-phospho-PDHA (Ser293) (1:1000, Millipore #AP1062),
Mouse anti-PDHA (1:1000, Invitrogen #45-6600),
Rabbit anti-p-Akt (Ser473) (1:1000, Cell Signaling Technology #9271),
Rabbit anti-Akt (1:3000, Cell Signaling Technology #9272),
Rabbit anti-p-mTOR (Ser2448) [D9C2] (1:1000, Cell Signaling Technology #5536),
Rabbit anti-mTOR [7C10] (1:3000, Cell Signaling Technology #2983),
Rabbit anti-p-S6 (Ser235/236) (1:3000, Cell Signaling Technology #4858),
Rabbit anti-S6 (1:3000, Cell Signaling Technology #2217),
Rabbit anti-p-AMPKα (Thr172) [40H9] (1:1000, Cell Signaling Technology #2535),
Rabbit anti-AMPKα (1:3000, Cell Signaling Technology #2532),
Mouse anti-β-actin [C4] (1:10000, Santa Cruz Biotechnology #sc-47778).
Mouse anti-MTCO1[1D6E1A8] (1:100, Abcam, #ab14705),

Mouse anti-Akt(pan)[40D4] (1:200, Cell Signaling Technology #2920),
and Rabbit anti-phospho-Akt(Ser473) (1:200, Abcam #ab81283).

Secondary antibodies:
Goat anti-Mouse IgG (H+L) Alexa Fluor 488 (Invitrogen #A11029)
Goat anti-Mouse IgG IRDye® 680RD (1:10,000, Li-COR Biosciences #926-68070),
Goat anti-Rabbit IgG IRDye® 800CWV (1:10,000, Li-COR Biosciences #926-32211).

Validation

All antibodies are commercial and validated by the manufacturer.
Primary antibodies:
Mouse anti-OxPhos antibody cocktail:
https://www.thermofisher.com/antibody/product/NDUFB8-Antibody-clone-20E9DH10C12-Monoclonal/459210
https://www.thermofisher.com/antibody/product/SDHB-Antibody-clone-21A11AE7-Monoclonal/459230
https://www.thermofisher.com/antibody/product/MTCO2-Antibody-clone-12C4F12-Monoclonal/A-6404
https://www.thermofisher.com/antibody/product/ATP5A1-Antibody-clone-15H4C4-Monoclonal/43-9800

Rabbit anti-SDHA [EPR9043(B)]: https://www.abcam.com/sdha-antibody-epr9043b-ab137040.html
Mouse anti-ATP5A [15H4C4]: https://www.abcam.com/atp5a-antibody-15h4c4-mitochondrial-marker-ab14748.html
Rabbit anti-PC: https://www.novusbio.com/products/pyruvate-carboxylase-antibody_nbp1-49536
Rabbit anti-phospho-PDHA (Ser293): https://www.merckmillipore.com/GB/en/product/PhosphoDetect-Anti-PDH-E1-pSer293-Rabbit-pAb,EMD_BIO-AP1062
Mouse anti-PDHA: https://www.thermofisher.com/antibody/product/PDHA1-Antibody-clone-8D10E6-Monoclonal/45-6600
Rabbit anti-p-Akt (Ser473): https://www.cellsignal.co.uk/products/primary-antibodies/phospho-akt-ser473-antibody/9271https://www.cellsignal.co.uk/products/primary-antibodies/phospho-akt-ser473-antibody/9271
Rabbit anti-Akt: https://www.cellsignal.co.uk/products/primary-antibodies/akt-antibody/9272
Rabbit anti-p-mTOR (Ser2448) [D9C2]: https://www.cellsignal.co.uk/products/primary-antibodies/phospho-mtor-ser2448-d9c2-xp-rabbit-mab/5536
Rabbit anti-mTOR [7C10]: https://www.cellsignal.co.uk/products/primary-antibodies/mtor-7c10-rabbit-mab/2983
Rabbit anti-p-S6 (Ser235/236): https://www.cellsignal.co.uk/products/primary-antibodies/phospho-s6-ribosomal-protein-ser235-236-d57-2-2e-xp-rabbit-mab/4858
Rabbit anti-S6: https://www.cellsignal.co.uk/products/primary-antibodies/s6-ribosomal-protein-5g10-rabbit-mab/2217
Rabbit anti-p-AMPKα (Thr172) [40H9]: https://www.cellsignal.co.uk/products/primary-antibodies/phospho-ampka-thr172-40h9-rabbit-mab/2535
Rabbit anti-AMPKα: https://www.cellsignal.co.uk/products/primary-antibodies/ampka-antibody/2532
Mouse anti-β-actin [C4]: https://www.scbt.com/p/beta-actin-antibody-c4?gclid=Cj0KCQjwwY-LBhD6ARIsACvT72OR3sd_f2PCjUef2jKXhoxkKlrfCesz0HrROg0JyMscnpAE5tP15DUaAr94EALw_wcB
Mouse anti-MTCO1[1D6E1A8]: https://www.abcam.com/mtco1-antibody-1d6e1a8-ab14705.html
Mouse anti-Akt(pan)[40D4]: https://www.cellsignal.co.uk/products/primary-antibodies/akt-pan-40d4-mouse-mab/2920
and Rabbit anti-phospho-Akt(Ser473): https://www.abcam.com/akt1-phospho-s473-antibody-ep2109y-ab81283.htmlhttps://www.abcam.com/akt1-phospho-s473-antibody-ep2109y-ab81283.html

Secondary antibodies:
Goat anti-Mouse IgG (H+L) Alexa Fluor 488: https://www.thermofisher.com/antibody/product/Goat-anti-Mouse-IgG-H-L-Highly-Cross-Adsorbed-Secondary-Antibody-Polyclonal/A-11029
Goat anti-Mouse IgG IRDye® 680RD: https://www.licor.com/bio/reagents/irdye-680rd-goat-anti-mouse-igg-secondary-antibody
Goat anti-Rabbit IgG IRDye® 800CWV: https://www.licor.com/bio/reagents/irdye-800cw-goat-anti-rabbit-igg-secondary-antibody

Eukaryotic cell lines

Policy information about cell lines

Cell line source(s)

Human fibroblasts (controls and patients) were from MRC CNMD Biobank London. A549 cybrid cells were a gift from Ian Holt (MRC National Institute for Medical Research, London). Also, 143B cybrid cells were shared by Michael Minczuk (MRC Mitochondrial Biology Unit, Cambridge, UK)

Authentication

None of the cell lines used were authenticated.

Mycoplasma contamination

All cell lines tested negative for mycoplasma.

Commonly misidentified lines
(See ICLAC register)

No commonly misidentified lines were used in this study.

Human research participants

Policy information about studies involving human research participants

Population characteristics

The muscle biopsies were from a female patient with the m.3243A>G mutation (at the age of 55) and a matched healthy control.

Recruitment

informed consent was obtained from all participants.

Ethics oversight | The study was approved by the Queen Square Research Ethics Committee, London (09/H0716/76).

Note that full information on the approval of the study protocol must also be provided in the manuscript.

Flow Cytometry

Plots

Confirm that:

- [x] The axis labels state the marker and fluorochrome used (e.g. CD4-FITC).
- [x] The axis scales are clearly visible. Include numbers along axes only for bottom left plot of group (a 'group' is an analysis of identical markers).
- [x] All plots are contour plots with outliers or pseudocolor plots.
- [x] A numerical value for number of cells or percentage (with statistics) is provided.

Methodology

Sample preparation	Cells were trypsinised and resuspended in PBS with 1% BSA
Instrument	BD Aria III
Software	BD FACSdiva 7
Cell population abundance	N/A, since we only separated debris from cells,which were then sorted for single cell PCR .
Gating strategy	For single cell sorting, cells were gated on FSC-A and SSC-A to exclude debris. The gating strategy is shown in Supplementary Fig.7 e-f.

- [x] Tick this box to confirm that a figure exemplifying the gating strategy is provided in the Supplementary Information.
