# Supplementary material for: Constitutive activation of the PI3K-Akt-mTORC1 pathway sustains the m.3243 A > G mtDNA mutation
Source: Nat Commun. 2021 Nov 4;12:6409. doi: 10.1038/s41467-021-26746-2 (PMC8568893; doi:10.1038/s41467-021-26746-2)
Supplement: Supplementary file 4 — Source Data [file 41467_2021_26746_MOESM4_ESM.zip › Source data/Source data_uncropped immunoblotting images.pdf]

Fig. 1a

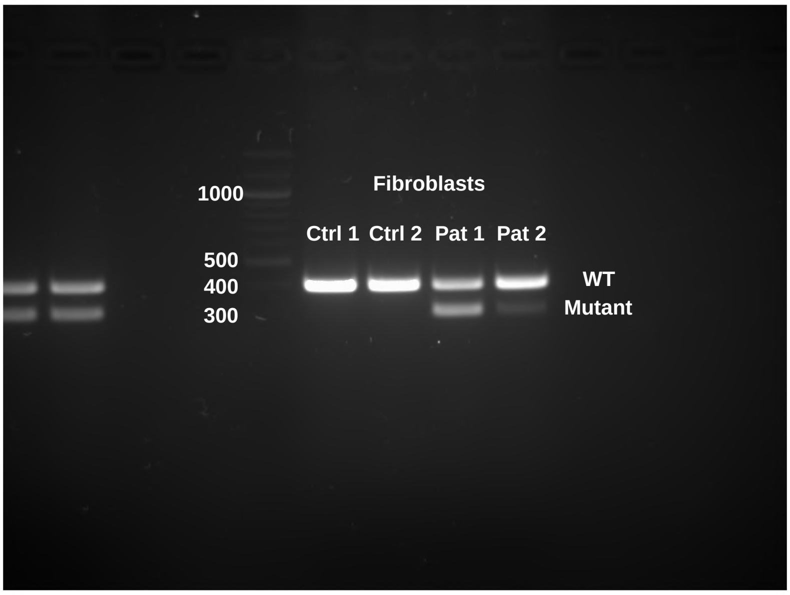

1000
500
400
300
Fibroblasts
Ctrl 1
Ctrl 2
Pat 1
Pat 2
WT
Mutant

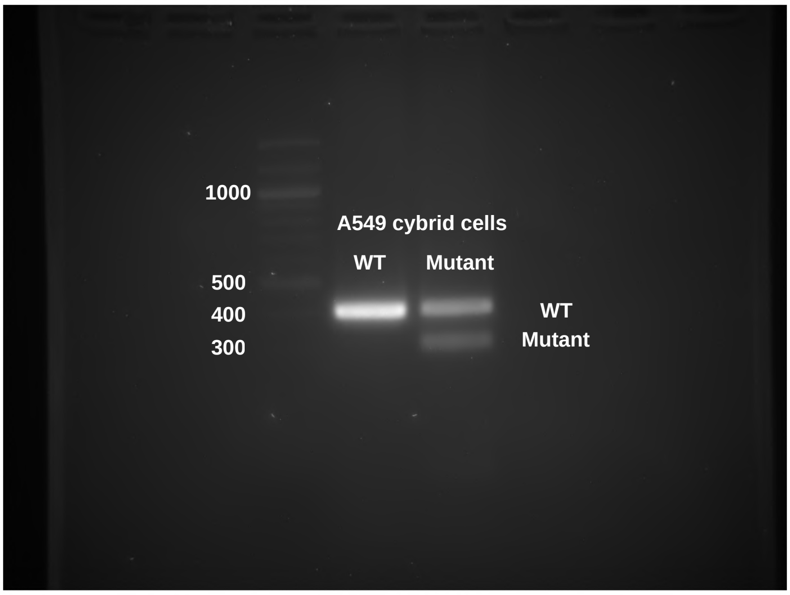

1000
500
400
300
A549 cybrid cells
WT
Mutant
WT
Mutant

Fig. 1g

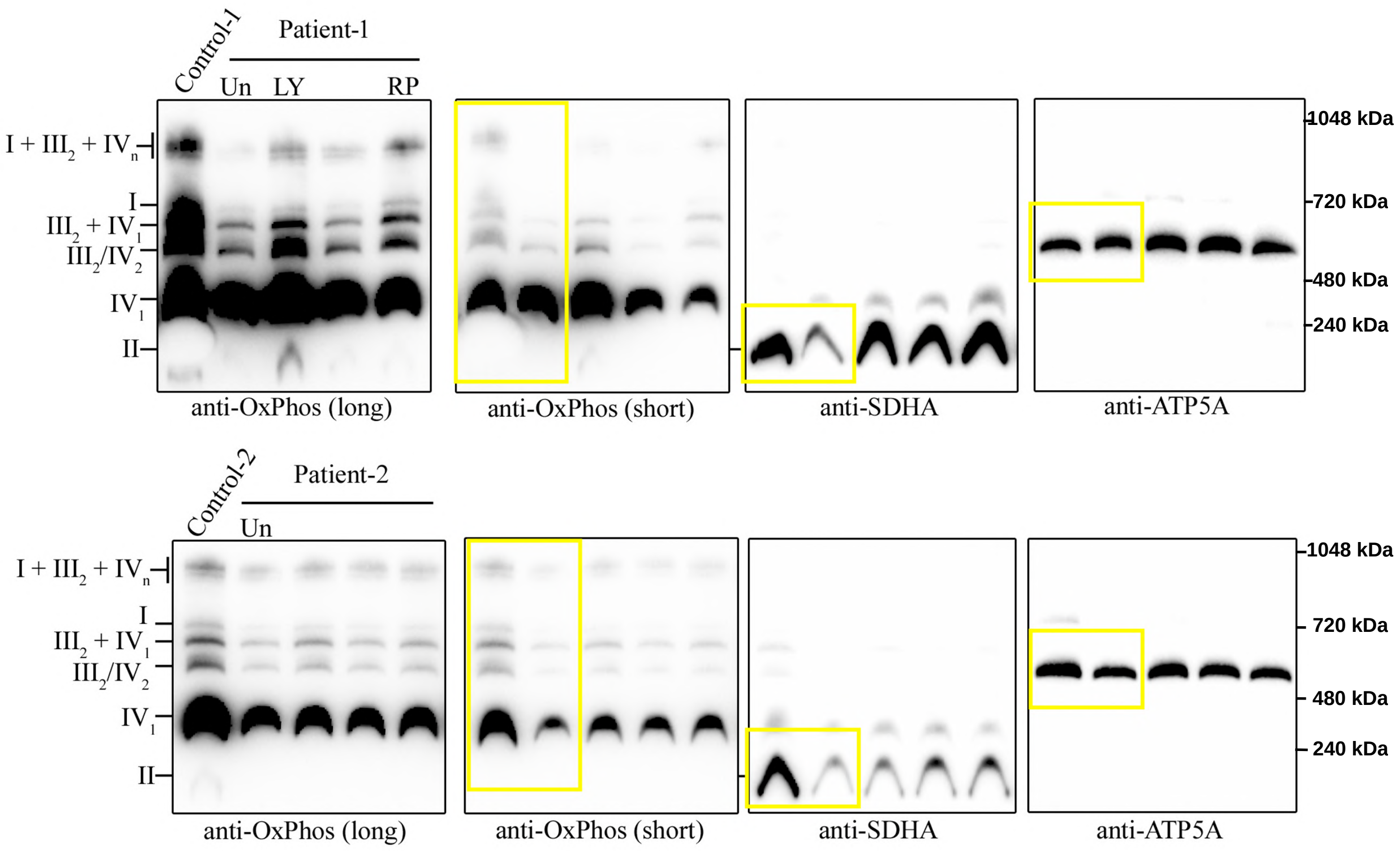

Control-1
Patient-1
Un
LY
RP
I + III2 + IVn
I
III2 + IV1
III2/IV2
IV1
II
anti-OxPhos (long)
anti-OxPhos (short)
anti-SDHA
anti-ATP5A
1048 kDa
720 kDa
480 kDa
240 kDa
Control-2
Patient-2
Un
I + III2 + IVn
I
III2 + IV1
III2/IV2
IV1
II
anti-OxPhos (long)
anti-OxPhos (short)
anti-SDHA
anti-ATP5A
1048 kDa
720 kDa
480 kDa
240 kDa

Fig. 4b

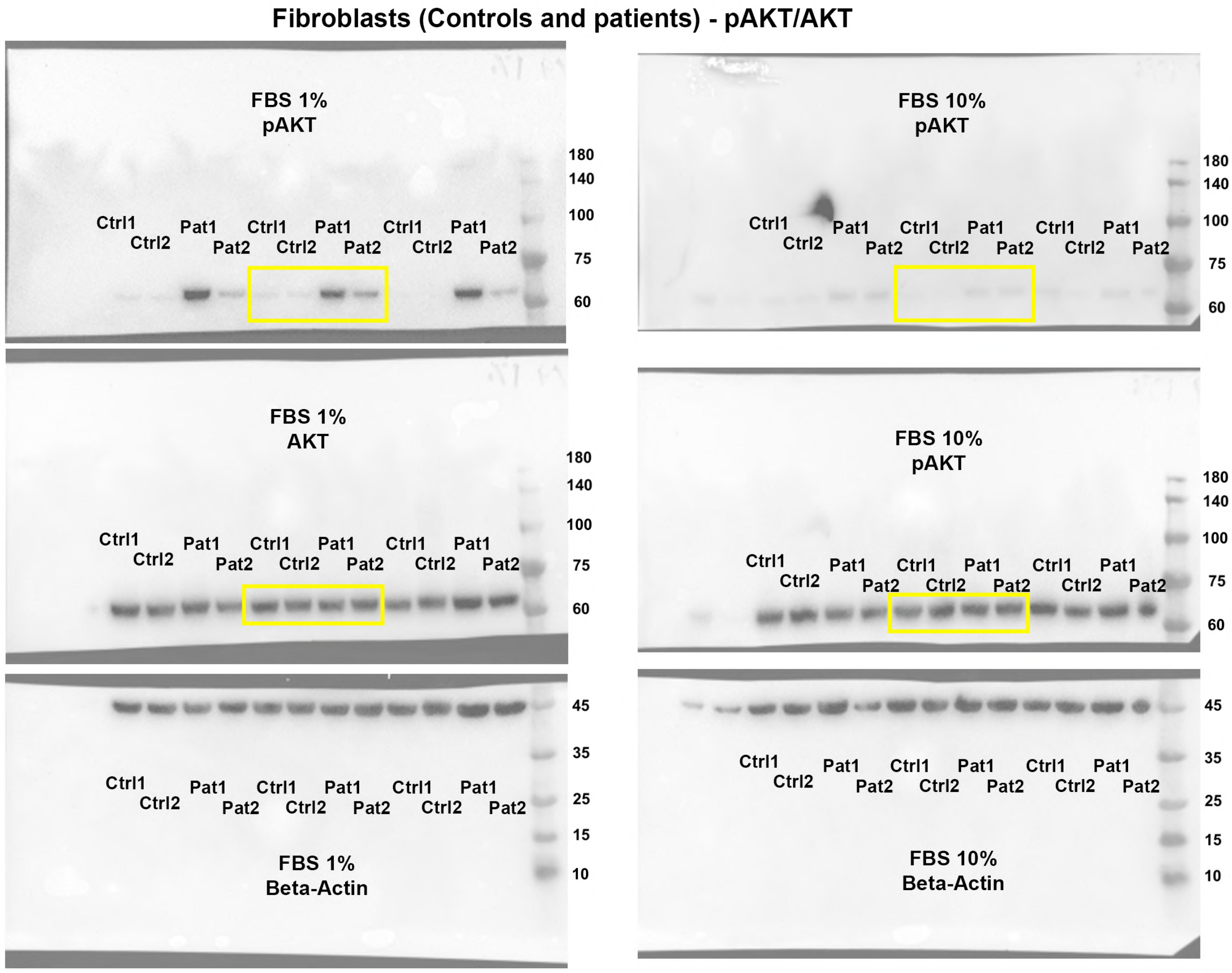
Fibroblasts (Controls and patients) - pAKT/AKT
FBS 1%
pAKT
Ctrl1 Ctrl2 Pat1 Pat2 Ctrl1 Ctrl2 Pat1 Pat2 Ctrl1 Ctrl2 Pat1 Pat2
180
140
100
75
60
FBS 10%
pAKT
Ctrl1 Ctrl2 Pat1 Pat2 Ctrl1 Ctrl2 Pat1 Pat2 Ctrl1 Ctrl2 Pat1 Pat2
180
140
100
75
60
FBS 1%
AKT
Ctrl1 Ctrl2 Pat1 Pat2 Ctrl1 Ctrl2 Pat1 Pat2 Ctrl1 Ctrl2 Pat1 Pat2
180
140
100
75
60
FBS 10%
pAKT
Ctrl1 Ctrl2 Pat1 Pat2 Ctrl1 Ctrl2 Pat1 Pat2 Ctrl1 Ctrl2 Pat1 Pat2
180
140
100
75
60
45
35
25
15
10
Ctrl1 Ctrl2 Pat1 Pat2 Ctrl1 Ctrl2 Pat1 Pat2 Ctrl1 Ctrl2 Pat1 Pat2
FBS 1%
Beta-Actin
45
35
25
15
10
Ctrl1 Ctrl2 Pat1 Pat2 Ctrl1 Ctrl2 Pat1 Pat2 Ctrl1 Ctrl2 Pat1 Pat2
FBS 10%
Beta-Actin

Fig. 4b

Fibroblasts (Controls and patients) - pS6/S6

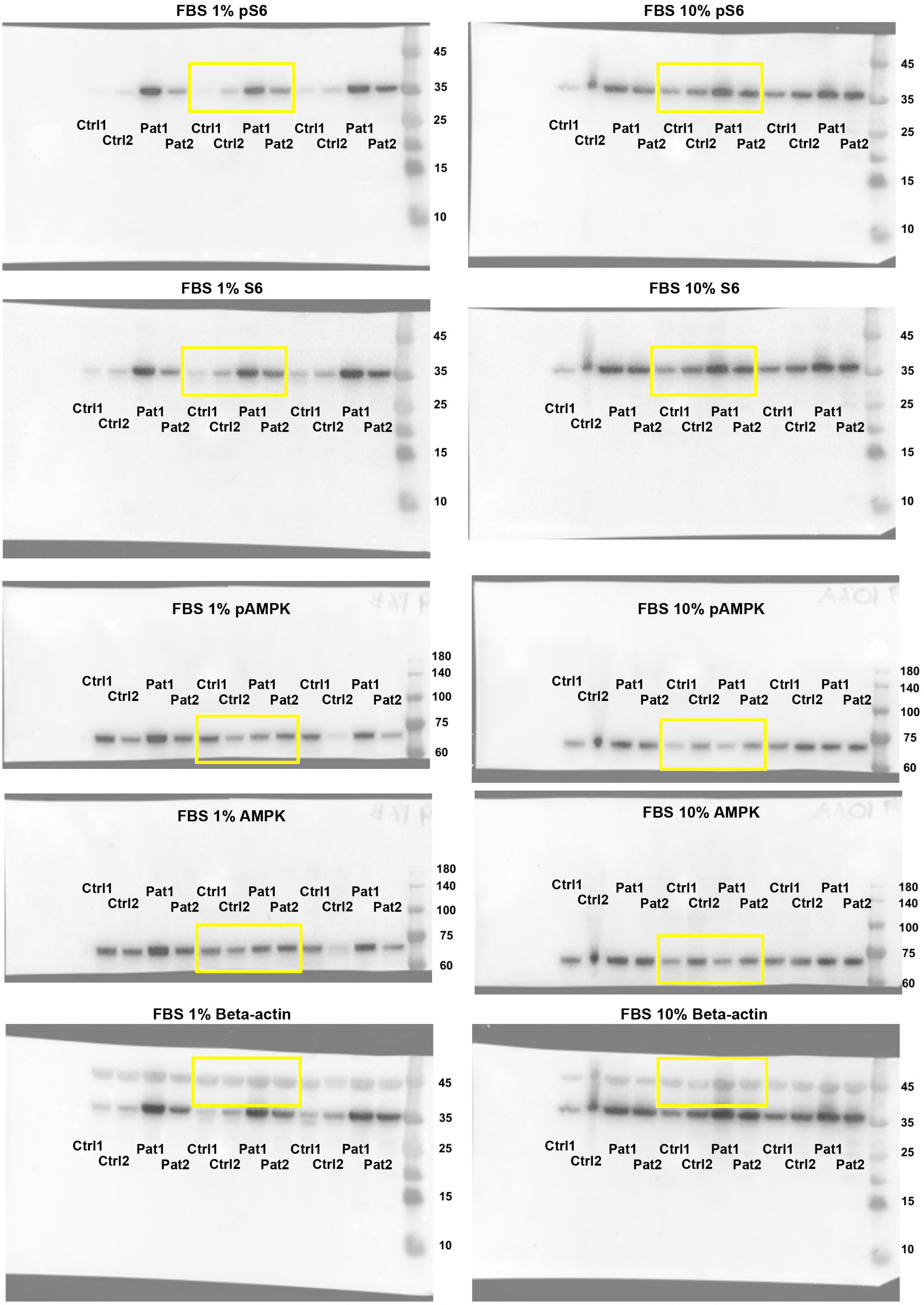

Fig. 5j

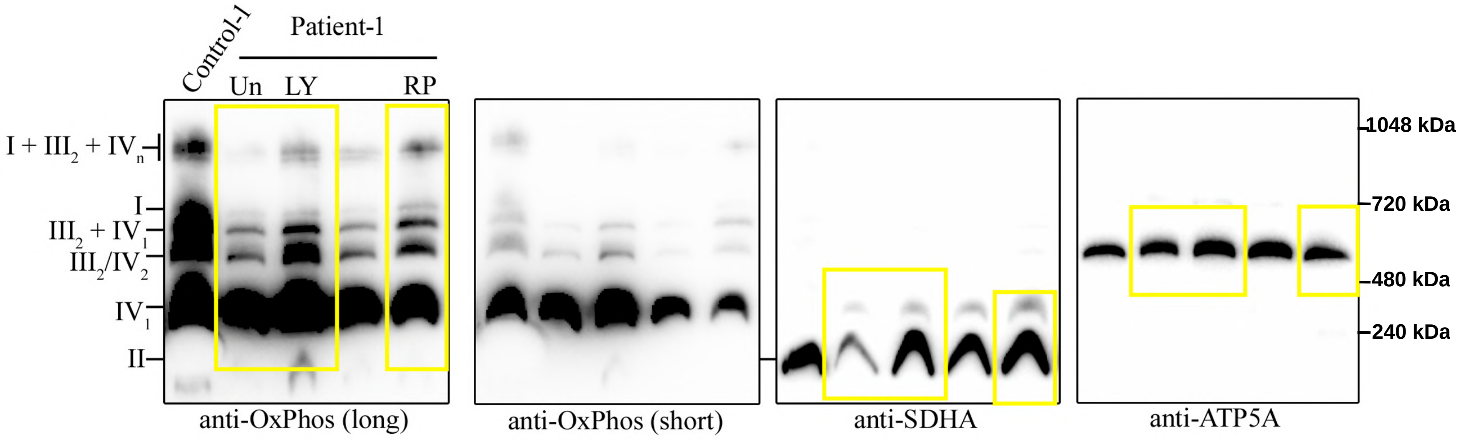

Control-1
Patient-1
Un
LY
RP
I + III_2 + IV_n
I
III_2 + IV_1
III_2/IV_2
IV_1
II
anti-OxPhos (long)
anti-OxPhos (short)
anti-SDHA
anti-ATP5A
1048 kDa
720 kDa
480 kDa
240 kDa

Supplementary Fig. 3d

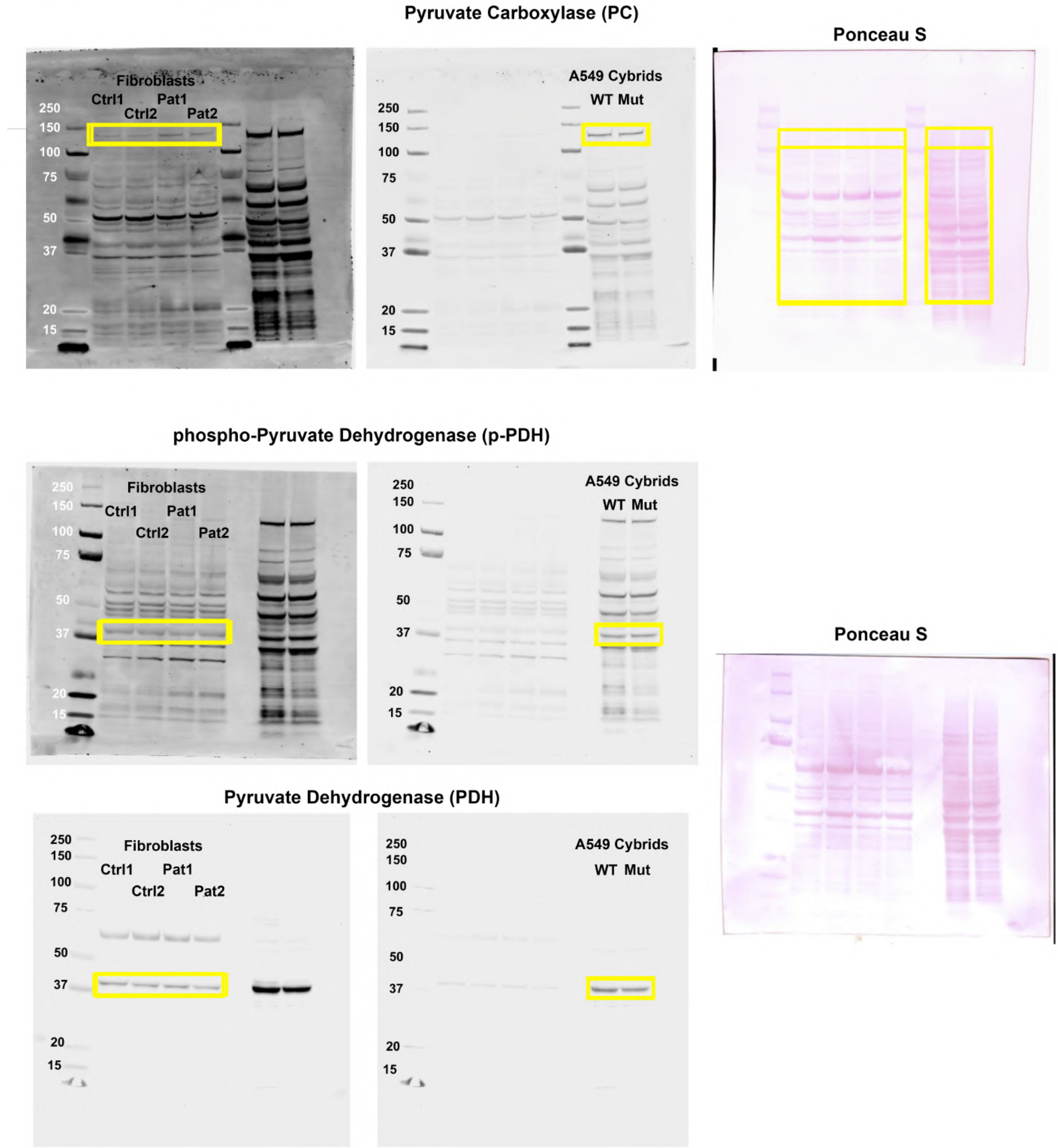

Supplementary Fig. 5a

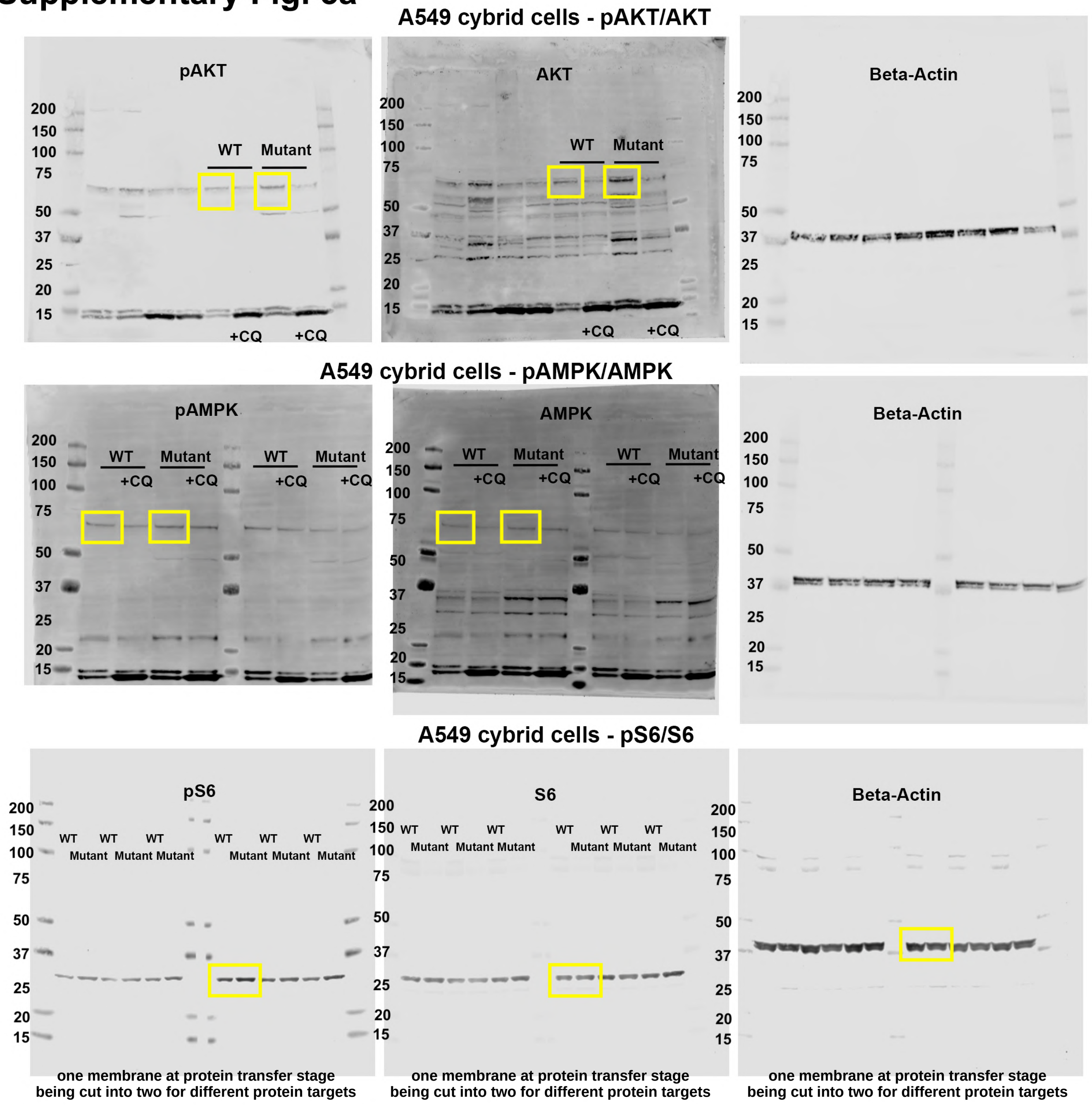

Supplementary Fig. 5c

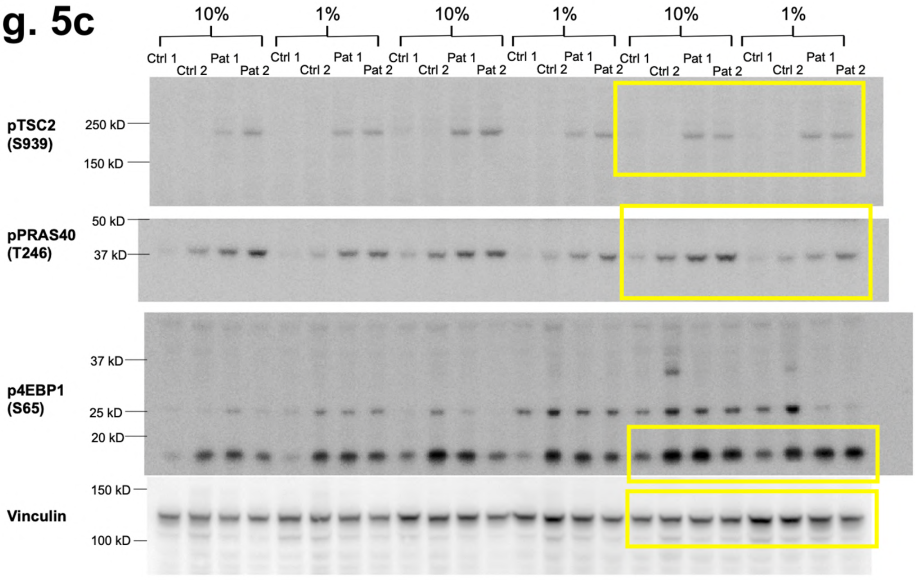

Supplementary Fig. 6a

A549 cybrid cells drug treatments (1-3 weeks) - pAMPK/AMPK, pAKT/AKT and p-mTOR/mTOR

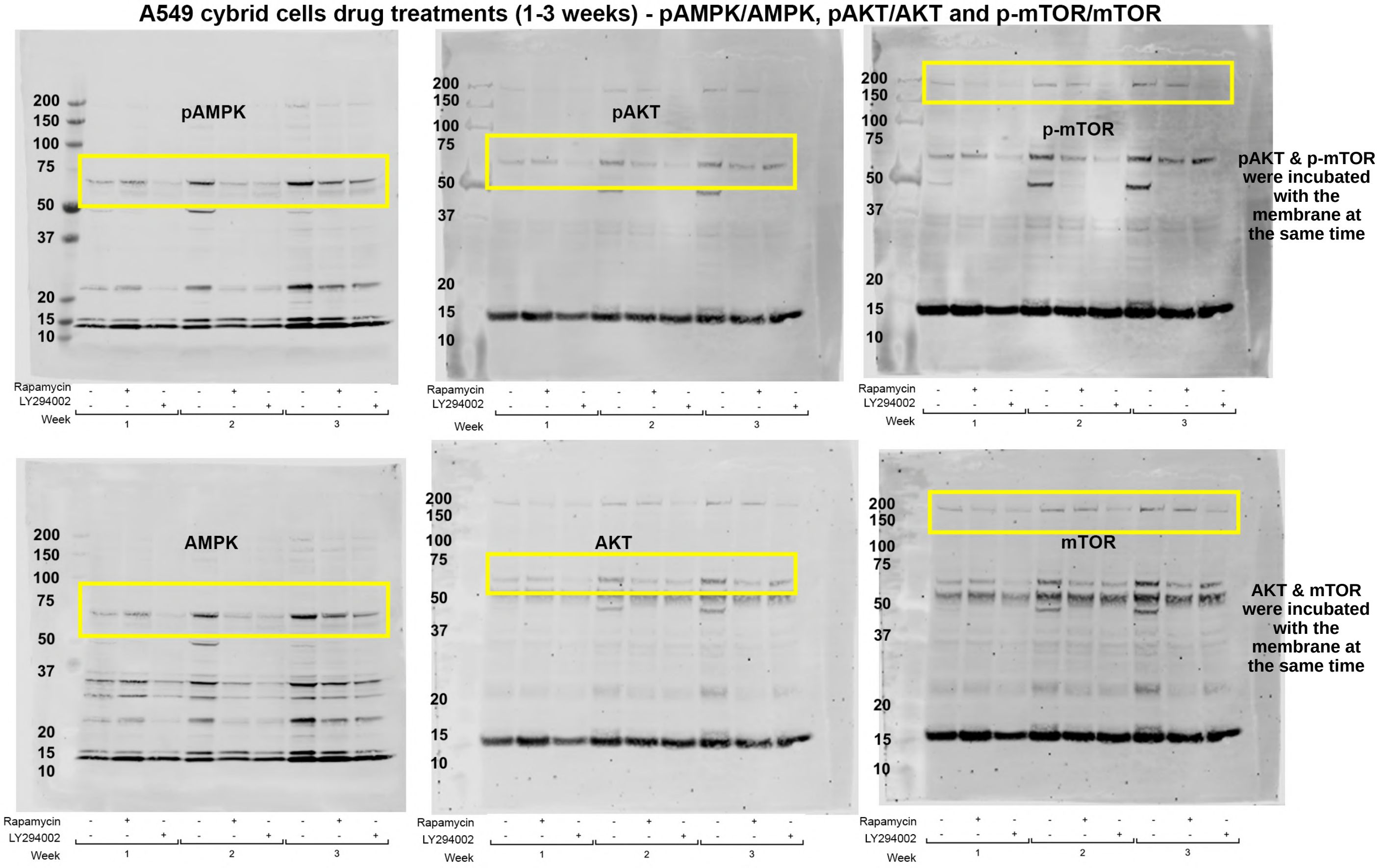

A549 cybrid cells drug treatments (6 weeks) - pAMPK/AMPK, pAKT/AKT and p-mTOR/mTOR

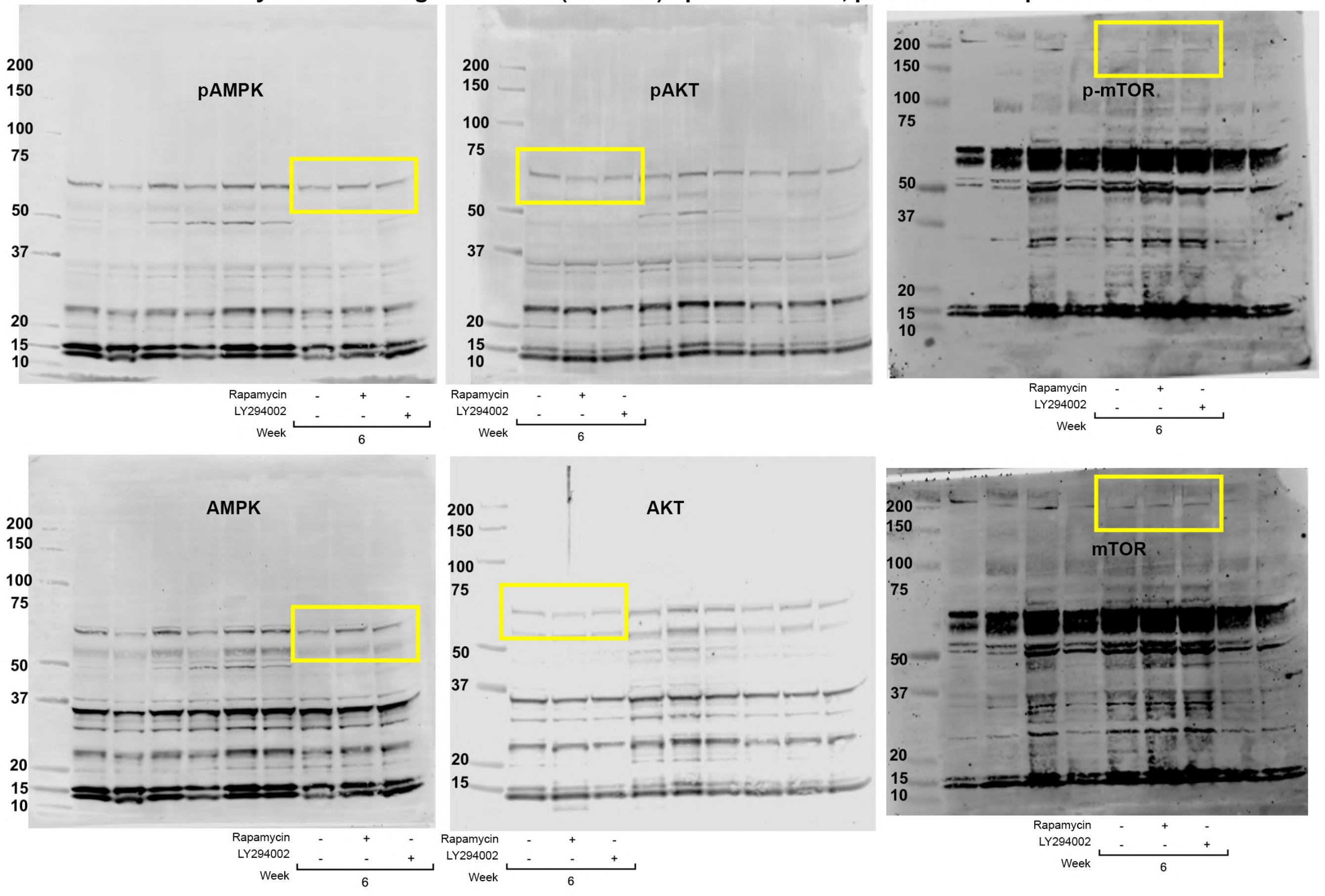

Supplementary Fig. 6d

Pat 1 Fibroblasts drug treatments (2-6 weeks) - pAMPK/AMPK, pAKT/AKT and pS6/S6

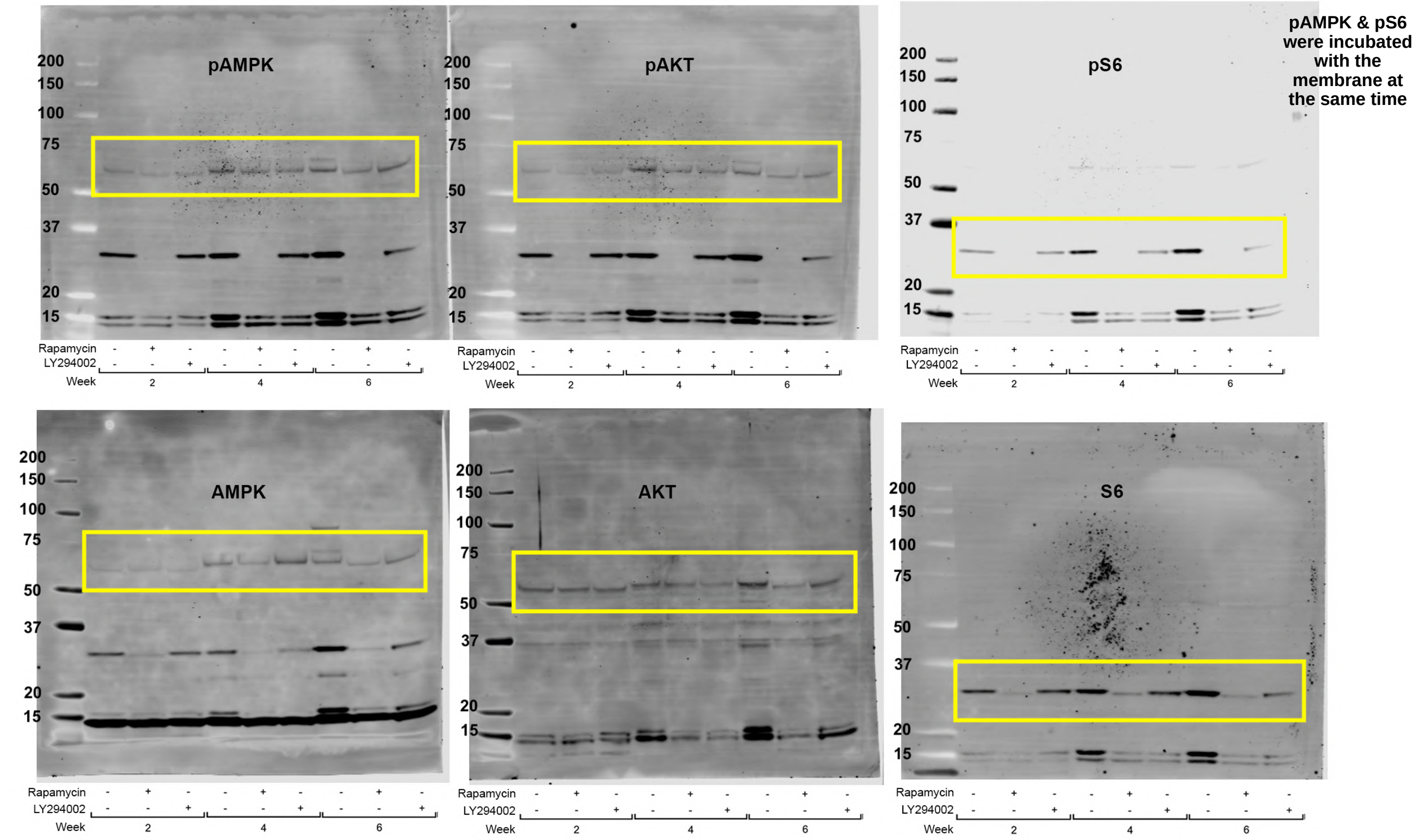

Pat 1 Fibroblasts drug treatments (12 weeks) - pAMPK/AMPK, pAKT/AKT and pS6/S6

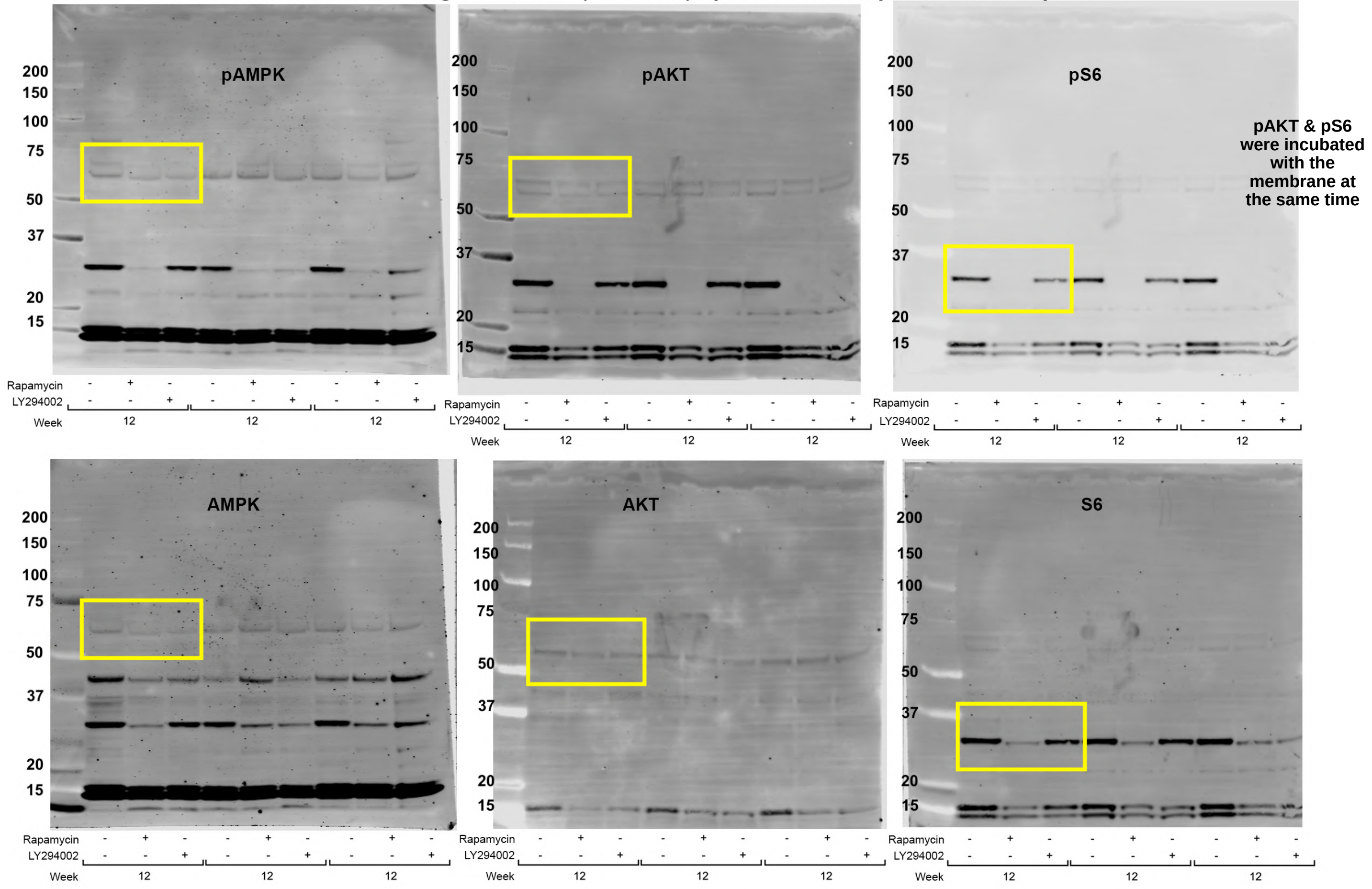

Supplementary Fig. 6g

A549 cybrid cells drug treatments - pS6/S6 and pAKT/AKT

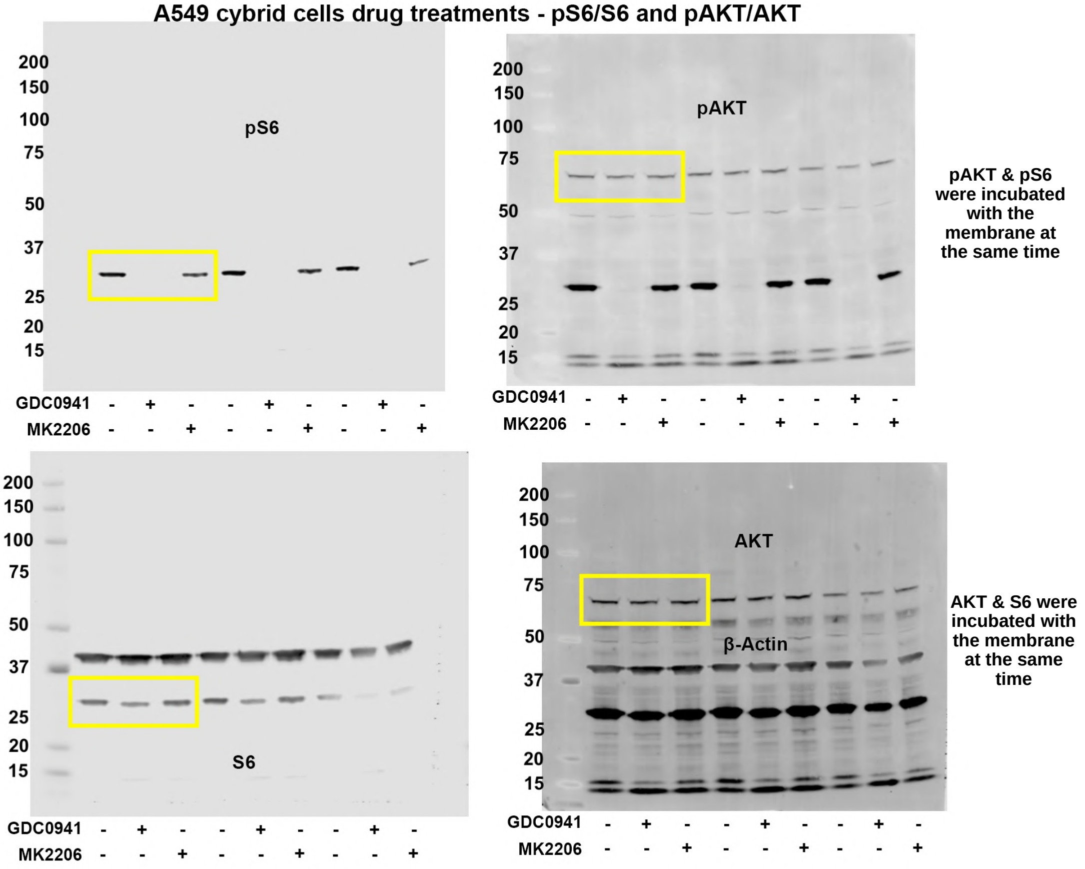

Pateint Fibroblasts drug treatments - pS6/S6 and pAKT/AKT

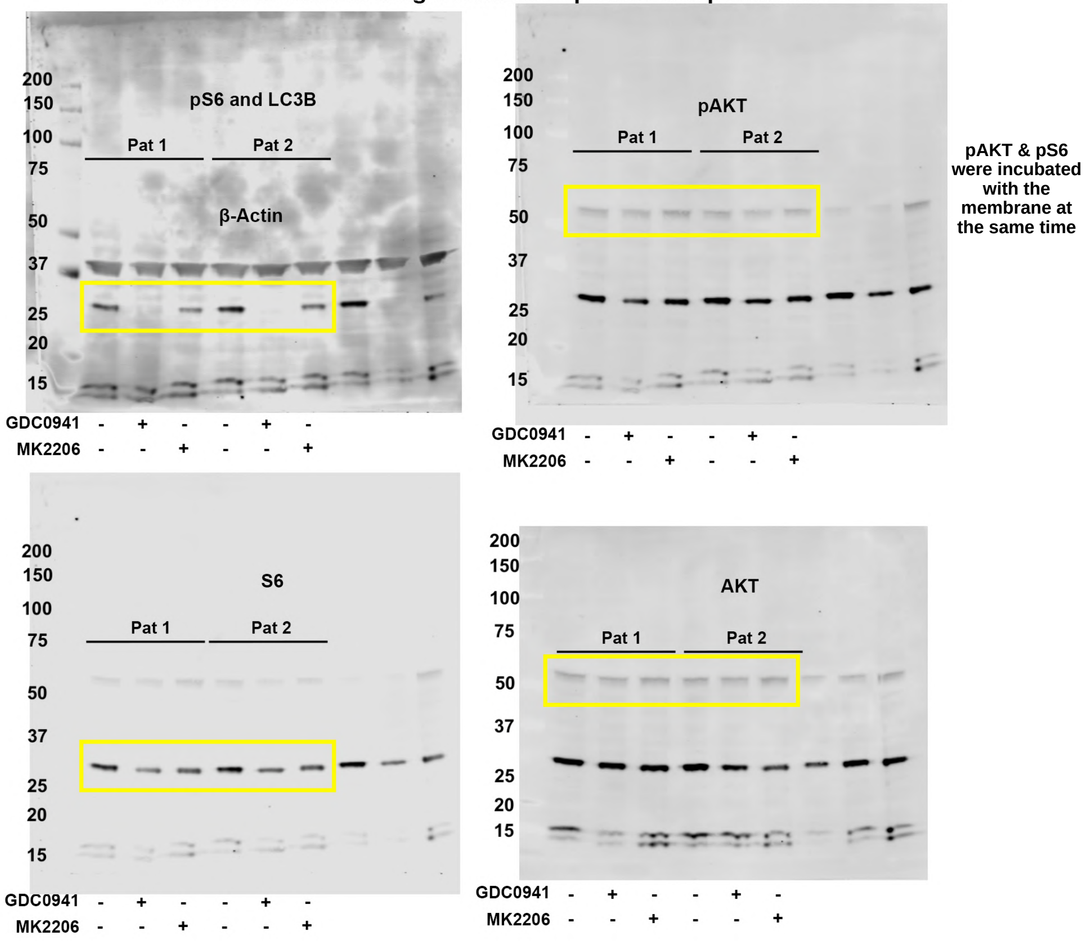

Supplementary Fig. 7h

T8993G 143B cybrid cells - pAKT/AKT, pS6/S6

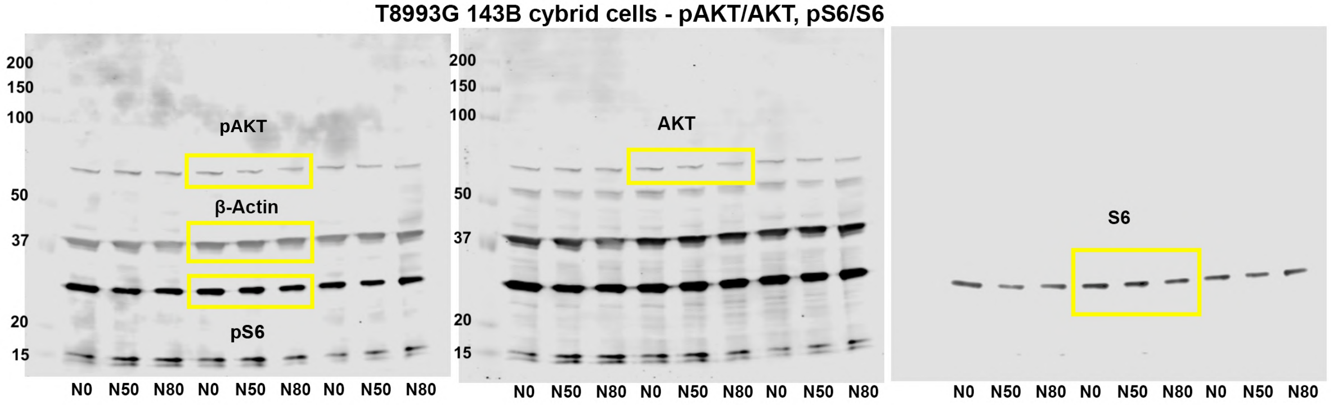

pAKT & pS6 were incubated with the membrane at the same time

AKT & S6 were incubated with the membrane at the same time
